# Green Sea Turtle Recruitment in the Eastern North Pacific: Patterns Identified Using Geochemical Signatures in Bones

**DOI:** 10.1002/ece3.72482

**Published:** 2026-01-13

**Authors:** Calandra N. Turner Tomaszewicz, Erin LaCasella, Garrett E. Lemons, Robin LeRoux, Jeffrey A. Seminoff

**Affiliations:** ^1^ NOAA, Southwest Fisheries Science Center La Jolla California USA

**Keywords:** habitat shift, life history, sea turtle, stable isotopes

## Abstract

Within marine systems, nutrient cycling is driven by physical forces that create predictable geochemical gradients. In turn, these gradients are reflected in spatially explicit and chemically distinct foodwebs, creating unique chemical signatures of consumer tissues that are useful for tracking the location and diet of consumers. In the eastern North Pacific, over the past three decades green sea turtles ( *Chelonia mydas* ) have become more commonly observed along the west coast of the United States, particularly along the urban Southern California coast. Understanding the habitat use patterns and basic demographic rates of these turtles is important for resource management. To address these data gaps, we used spatial patterns created by natural geochemical cycling (i.e., marine isoscapes) to inform sea turtle movement and habitat use over time. This was done by analyzing stable isotope values of bone growth layers in turtle humeri and analyzing the values with age and size data obtained through skeletochronology. This approach allowed us to recreate the movements and foraging patterns of green sea turtles in Southern California. We present vital life‐history and demographic data, including the oceanic stage duration, timing of ontogenetic habitat shifts, and multi‐year foraging patterns. Sea turtles depart the oceanic habitat recruiting to neritic foraging grounds around 6.6 years of age, indicated by nitrogen isotope values (δ^15^N), but turtles may do so as early as one year old, or may remain in oceanic zones for much longer. Once settled into isotopically distinct coastal habitats, it was common for turtles to establish multi‐year residency, and while many appeared to consume at least some seagrass, stable carbon isotope values (δ^13^C)—a primary indicator of critical habitat—suggested that it was not the primary diet item of most individuals. Collectively, these findings fill information gaps about green turtle life‐history, which have immediate application to ongoing regional management efforts.

## Graphical abstract

We present vital life‐history and demographic data including the oceanic stage duration, timing of ontogenetic habitat shifts, and multi‐year foraging patterns of eastern North Pacific green sea turtles found in Southern California. The findings were obtained by analyzing sea turtle bones (age and growth via skeletochronology; and habitat and diet via stable isotope analysis) in conjunction with data obtained from in‐water monitoring, and provide detailed, multi‐year information on individual green sea turtles.

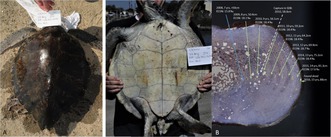

## 1 Introduction

**FIGURE 1:**
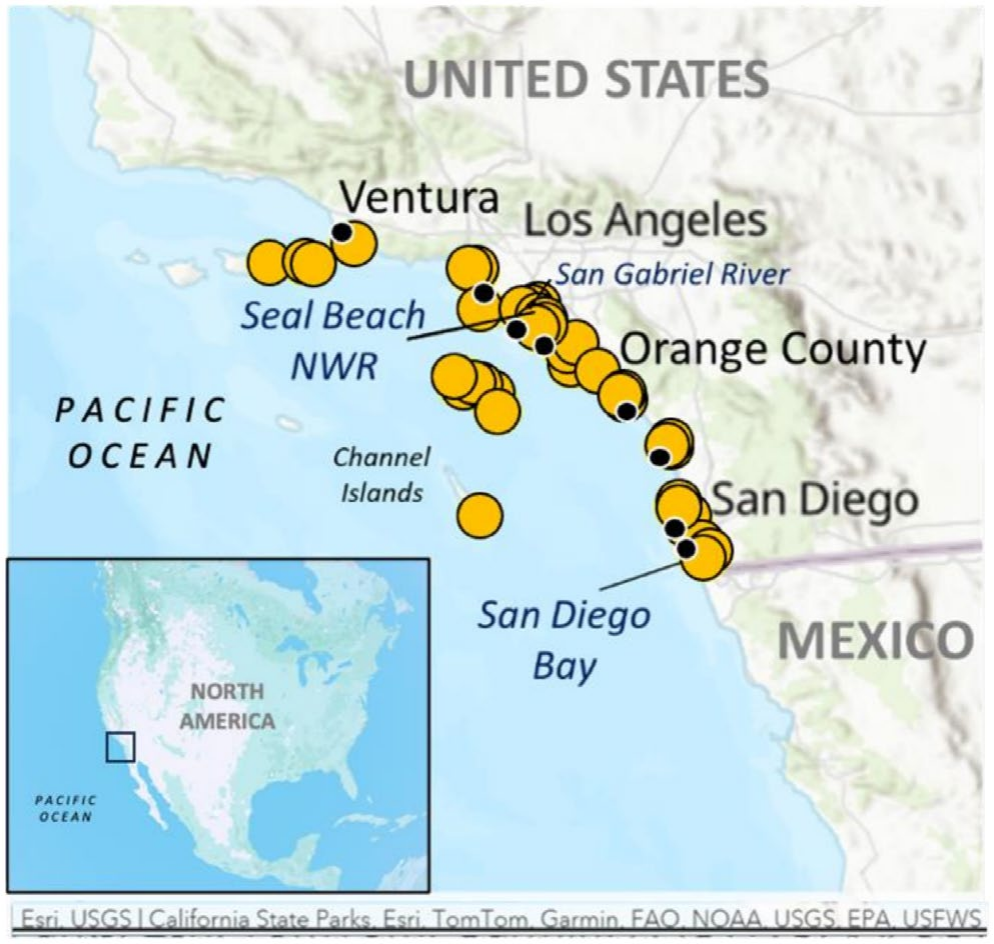
Green sea turtle sightings (yellow circles with yellow outline) reported by community members to the SWFSC ArcGIS survey at https://tinyurl.com/SeeASeaTurtle since 2022, and locations where dead sea turtles (black circles) were recovered and humerus bones were then analyzed for the current study. Locations of the 39 turtles were as follows: 31 in San Diego County, eight in Orange County, and one each along the coast in Los Angeles and Ventura County. Inset map shows the study area at a larger scale.

In the eastern North Pacific (ENP), green sea turtles ( *Chelonia mydas* ) have shown remarkable recovery from the risk of extinction since the 1990s (NOAA NMFS 2015; Bedolla‐Ortiz et al. 2023). Protection efforts at the nesting beaches in Pacific Mexico and the cessation of the hunting and international trade of turtles captured in northwest Mexico are the main reasons for this rebound (NOAA NMFS 2015; Early‐Capistrán et al. 2022; Bedolla‐Ortiz et al. 2023). The known primary nesting beaches in the northern range of this population are in Mexico's state of Michoacán, and the islands of the Revillagigedo and Tres Marias Archipelagos (Dutton et al. 2014, 2019; NOAA NMFS 2015) (Figure 1). During their juvenile stage and during breeding migrations, these green sea turtles occupy oceanic offshore waters in the eastern Pacific (NOAA NMFS 2015) (Figure 1). Once the turtles grow and depart the oceanic habitat, their nearshore foraging grounds include northern areas along the coasts of mainland and islands of Mexico, including the Baja California Peninsula, as well as Southern California in the United States (US; Senko et al. 2010; NOAA NMFS 2015; Dutton et al. 2019). In the US, west coast green sea turtle foraging aggregations are well documented, and in recent years, turtles have been sighted throughout diverse habitats along the coast, including bays and lagoons of southern California and the Channel Islands (Hanna et al. 2021; Seminoff et al. 2021; Massey et al. 2023; NOAA NMFS, unpublished manuscript). Current understanding of movement and habitat use of green sea turtles in the Southern California region comes primarily from flipper and satellite tagging, as well as from community‐based sightings (MacDonald et al. 2013; Massey et al. 2023; Maurer et al. 2024; NOAA NMFS; Sea Turtle Survey https://tinyurl.com/SeeASeaTurtle). Today, green sea turtles from this recovering population are a regular occurrence off the US West Coast, with over 600 sightings reported by community members since 2022 (NOAA NMFS Sea Turtle Survey, Figure 1). The increased presence of sea turtles near highly urban areas and coastal fishing sites reinforces the need to better understand the movement and habitat use of these turtles in areas along the US West Coast.

In Southern California, green sea turtles have been studied during in‐water research at three different locations: the San Diego Bay (SDB) studied since 1990, the San Gabriel River (SGR) studied since 2010, and the Seal Beach National Wildlife Refuge (SBNWR) studied since 2014. Collectively, hundreds of green sea turtles have been captured, tagged, measured, and sampled to inform population abundance, health, ecology, and demography (Eguchi et al. 2012; Allen et al. 2015; Dutton et al. 2019; Seminoff et al. 2021; Maurer et al. 2024; NOAA NMFS, unpublished manuscript). In addition to the study of live animals, much has also been learned by analyzing samples collected from dead stranded turtles recovered along the US West Coast; this sample collection is possible through the efforts of a large regional network of partnering organizations and agencies. Samples collected from these dead stranded animals have informed the demographics of green sea turtles along the US West Coast, where Turner Tomaszewicz, Avens, et al. (2022) documented the wide variability in the age and growth rates of green sea turtles within this region's foraging population, and provided empirical data for the age and size at maturation. Yet key gaps remain in the understanding of the habitat use patterns and basic demographic rates of these turtles in Southern California waters, and addressing these gaps is important for effective resource management.

By leveraging existing biogeochemical spatial patterns, it is possible to efficiently and informatively infer the location and/or diet of animals over time when analyzing their stable isotope values (Hobson 1999). This method has become particularly useful for studying long‐lived species that occupy remote habitats and are challenging to access and study (McMahon et al. 2013; Truman and St. John Glew 2019; Vander Zanden et al. forthcoming). The natural cycling of water and nutrients (e.g., nitrogen, phosphorus, carbon, sulfur) in various ecosystems, both terrestrial and aquatic, creates predictable geochemical gradients (Hobson 1999). Within ocean systems, such geochemical gradients are the result of various processes such as nutrient availability and corresponding productivity, and ocean circulation patterns such as upwelling (Wada et al. 1987; Deutsch et al. 2001; Altabet 2006; Castro et al. 2020). The resulting unique isotopic patterns are often visualized as “isoscapes” (spatial maps of isotopic variation) or characterized as specific “isotopic signatures” (like fingerprints), which can be used as diagnostic markers to distinguish between different, specific ocean habitats such as pelagic vs. benthic, or oceanic vs. neritic (Deutsch and Voss 2006; Magozzi et al. 2017; Vander Zanden et al. forthcoming). In animal biology, this becomes incredibly useful. When animals consume food and water from these isotopically characterized habitats, their tissues incorporate and reflect these unique isotopic signatures. By analyzing the stable isotope (SI) values (e.g., carbon, nitrogen, oxygen, hydrogen) in animal tissues, the SI values can then be matched to the known isotopic patterns of specific ocean habitats (Bowen 2010; Rossman et al. 2016). This powerful technique allows for the tracking (or recreating) of animal movement patterns, including long‐distance migrations, and shifts in habitat use throughout an animal's development (ontogenetic habitat shifts) (in Hobson and Wassenaar 2019—*terrestrial* Bowen and West 2019; *marine* Truman and St. John Glew 2019).

The recreation of habitat use over time is done by sequentially sampling stable isotopes from the layers of accretionary tissues, that is, tissues that grow in layers at regular time intervals—such as teeth, bones, shells, and feathers (Newsome et al. 2010; Hobson and Wassenaar 2019; Trofimova et al. 2020). Then, the SI values are matched to the isotopically characterized habitats, thus assigning the animal to a particular habitat for each corresponding growth layer. Altogether, the multiple sequential samples reflect that animal's movement over time, showing when a habitat shift occurred, and providing information about habitat residency duration. This record may span different periods of time—months, years, or even decades—depending on the tissue used and its corresponding rate of formation (Koch 1998; Newsome et al. 2010; Hobson and Wassenaar 2019). Sea turtles are one example of a long‐lived taxon that travels vast distances, and in doing so, integrates biogeochemical signatures into their tissues that are reflective of disparate habitats. Thus, by analyzing accretionary tissues of turtles that have passed through different ocean habitats across geochemical gradients, the patterns of their movements across isotopically distinct locations are revealed (e.g., Vander Zanden et al. forthcoming; Turner Tomaszewicz et al. 2015).

For the six species of hard‐shelled sea turtles, their humeri retain annual growth layers and provide an inert tissue ideal for sequential sampling for stable isotope analysis (Snover et al. 2010; Turner Tomaszewicz and Avens forthcoming). This technique, called ‘skeleto+iso’ (Turner Tomaszewicz et al. 2016; Turner Tomaszewicz, Liles, et al. 2022), has proven effective for recreating the multi‐year habitat‐use patterns and long‐term foraging behavior of several sea turtle populations (East Pacific greens Turner Tomaszewicz et al. 2018; North Pacific loggerheads *Caretta caretta* Turner Tomaszewicz, Seminoff, Peckham, et al. 2017; West Atlantic loggerheads Ramirez et al. 2015; East Pacific hawksbills *Eretmochelys imbricata* Turner Tomaszewicz, Liles, et al. 2022; Kemp's ridleys *Lepidochelys kempii* Avens et al. (2020); flatbacks *Natador depressus* Cahill et al., in preparation, Pacific olive ridleys *Lepidochelys oliviacea* Turner Tomaszewicz et al. in preparation). The current study aims to build upon this earlier work by using previously aged bones from Turner Tomaszewicz, Avens, et al. (2022), and applying complementary sequential stable isotope analysis of individual bone growth layers to examine patterns in habitat use and diet to further improve our understanding of sea turtle demography and movement.

In the ENP, established geochemical gradients make skeleto+iso a useful tool. In particular, stable nitrogen isotopes (δ^15^N) have well‐documented spatial gradients that allow for the isotopic characterization of habitats that are used to identify when turtles are using different locations (Olson et al. 2010; Somes et al. 2010; Ryabenko 2013; Allen et al. 2013; Turner Tomaszewicz, Seminoff, Peckham, et al. 2017). Broadly, there are distinct nitrogen isotope patterns that distinguish between the Central North Pacific (CNP) pelagic, the ENP pelagic, and the ENP neritic habitats (Somes et al. 2010). This is largely driven by nitrogen fixation in the CNP, producing lower δ^15^N values in comparison to denitrification in coastal upwelling in the ENP, creating higher δ^15^N values (Olson et al. 2010; Somes et al. 2010; Ryabenko 2013; Allen et al. 2013). Further ^15^N enrichment occurs in benthic habitat foodwebs, allowing for the greater distinction between consumers foraging in pelagic vs. neritic benthic foodwebs. Stable carbon isotope values (δ^13^C) are also useful in distinguishing specific habitats and or food items for green sea turtles in the ENP. In general, δ^13^C values decrease from seagrasses, to marine algae, to freshwater input macrophytes and periphyton (Clementz and Koch 2001; Camilleri and Ozersky 2019). This pattern exists because seagrass (an angiosperm) and algae each have unique photosynthetic pathways such that seagrasses have higher δ^13^C values (Hemminga and Mateo 1996; Clementz and Koch 2001; Ben‐David and Flaherty 2012); and in habitats with freshwater and terrestrial inputs, high algal growth and the presence of periphyton (floating and submerged filamentous mats of algae, cyanobacteria, microbes and detritus) produces δ^13^C values that are even lower than those of fully marine systems (Clementz and Koch 2001; Camilleri and Ozersky 2019).

The current study aims to better understand green sea turtle demography, movements, and habitat use patterns in Southern California, US. Specifically, knowledge about where turtles spend time, and for how long turtles occupy specific habitats (residency duration) is needed. This is a timely issue because, as this population's numbers continue to increase, it is expected that in Southern California, there will be a continued rise in overlap and interaction with human activities. Here, to recreate the movement patterns and diet of green sea turtles in Southern California, the current study builds off the previous skeletochronology work (Turner Tomaszewicz, Avens, et al. 2022) and adds complementary SI analysis of the same bones' growth layers, using the skelto+iso technique. We used the results to (1) estimate the oceanic stage duration of juvenile green sea turtles in the eastern North Pacific by estimating the age and size at departure from the oceanic habitat and settlement into coastal waters near the US West Coast; and (2) examine the long‐term foraging patterns once turtles move into a coastal Southern California habitat. To further interpret our SI findings, we used additional information, when possible, reported during in‐water capture efforts and post‐mortem necropsies to further inform the location and diet of individual turtles.

## 2 Methods

### 2.1 Sample Collection

We used 39 green sea turtles' bones previously processed and analyzed for age and growth estimation by skeletochronological analysis (Turner Tomaszewicz, Avens, et al. 2022). Full details are described in the previous study, but briefly, the bones had been collected from dead‐stranded green sea turtles recovered in Southern California (Figure 1), and were collected under NMFS Permits #s 1591, 14510, 16,803, 18,238, 28,119. The turtles used in the current study (a subset of those previously aged) spanned the full range of body sizes and ages of turtles included in the previous study, Turner Tomaszewicz, Avens, et al. (2022). The 39 turtles in the current study had body sizes ranging from 43 to 110.5 cm CCL (mean ± SD, 68.6 ± 17.6), and estimated final ages between five and 50 years old (14.0 ± 11.8 years SD; Table 1; Turner Tomaszewicz, Avens, et al. 2022).

**TABLE 1:** Estimated age and body size (curved carapace length, CCL) at stranding for all 39 turtles, by stranding location.

Location	No. turtles	CCL (cm)			Estimated age (years)		
		Mean ± SD	Range	SE	Mean ± SD	Range	SE
All	39	68.6 ± 17.6	43–110	2.8	14 ± 11.8	5–50	1.9
Los Angeles/Ventura Counties	2	62.5 ± 12.0	54–71	8.5	13.5 ± 10.6	6–21	7.5
Orange County	7	72.4 ± 17.9	49–99	6.8	15.3 ± 12.0	6–33	4.54
San Diego County	30	68.2 ± 18.1	43–110	3.3	13.7 ± 12.2	5–50	2.23

### 2.2 Turtle Bone Stable Isotope Analysis

From each bone cross‐section, we used a computer‐guided micromill (Carpenter Microsystems CM‐2 version 3.0.6) to extract ~1.5 mg of bone powder from individual annual growth layers for stable isotope analysis, which was then collected and packed into tin capsules and weighed using a Sartorius Microbalance, as fully described in Turner Tomaszewicz et al. (2016) and Turner Tomaszewicz, Seminoff, Price, and Kurle (2017). In brief, we used the identified lines of arrested growth (LAGs) in the skeletochronology‐derived images to guide precision micromill sampling from each growth layer. Samples were sent to the University of Florida, Gainesville, Florida, US, for stable isotope analyses of nitrogen (δ^15^N) and carbon (δ^13^C).

The tin capsules were placed in a Carlo Erba NA1500 CNS elemental analyzer. After combustion and separation of N_2_ from CO_2_, the sample gas was then passed into a ConFlo II preparation system and into the inlet of a Thermo Electron Delta V Advantage isotope ratio mass spectrometer running in continuous flow mode. Sample gas was measured relative to laboratory reference N_2_ and CO_2_ gases with all carbon isotopic results expressed in standard delta notation relative to Vienna Pee Dee Belemnite (VPDB), and all nitrogen isotopic results expressed in standard delta notation relative to air. Sample stable isotope ratios relative to the isotope standard are expressed in the following conventional delta (*δ*) notation in parts per thousand (‰): *δ* = ([*R*_sample_/*R*_standard_] – 1) × (1000), where *R*_sample_ and *R*_standard_ are the corresponding ratios of heavy to light isotopes (e.g., ^15^N/^14^N, ^13^C/^12^C) in the sample and standard, respectively. For all analytical runs, internationally recognized standard material samples (USGS40 and USGS41 from the USGS) with known δ^13^C and δ^15^N ratios were inserted every 6 to 7 samples to calibrate the system and compensate for any potential drift over time. Replicate assays (*n* = 87) of reference materials indicated measurement errors of 0.10‰ and 0.14‰ for carbon and nitrogen, respectively. The ratio of elemental concentrations of carbon and nitrogen (C:N) was used as quality assurance to assess stable isotope ratios for bone collagen (C:N < 3.5) as recommended and applied in previous cortical bone studies showing that the organic protein C:N ratio typically falls between 2.8 and 3.5 (Post et al. 2007; Turner Tomaszewicz et al. 2015). Finally, to account for the small amount of inorganic carbon in cortical bone of sea turtles, the carbon values (δ^13^C) were corrected using the experimentally derived correction equation for Pacific green sea turtles from Turner Tomaszewicz et al. (2016):

$$\delta^{13}C_{\mathrm{cor}}=\left(1.2\times \delta^{13}C_{\mathrm{raw}}\right)+2.1$$

where δ^13^C_cor_ is the corrected δ^13^C bone value and δ^13^C_raw_ is the raw, untreated, bone δ^13^C value. All stable carbon values reported herein as “δ^13^C” refer to corrected δ^13^C_cor_ values.

### 2.3 Characterizing Habitats Isotopically

To identify when green sea turtles departed oceanic habitats and moved into more nearshore neritic habitats of the ENP, we first characterized the isotopic values for the two habitats, oceanic and coastal, using previously published studies with spatially specific sea turtle reference samples—this is described in full detail below. Previous studies using a similar approach to characterize habitats isotopically have used the enriched stable nitrogen (high δ^15^N values) as a reliable identifier for nearshore coastal habitats in the eastern North Pacific when compared to oceanic habitats of the North Pacific (Allen et al. 2013; Turner Tomaszewicz, Seminoff, Peckham, et al. 2017; Turner Tomaszewicz, Liles, et al. 2022). In the current study, once the oceanic and coastal habitats were isotopically characterized based on the values obtained from the literature, we identified an oceanic‐departure threshold δ^15^N value to delineate the two habitats. Next, we used the threshold δ^15^N value to assign individual growth layers sampled for stable isotope analysis to one of the two habitats, oceanic or coastal. Altogether, this allowed for the recreation of movements of each turtle between the isotopically distinct habitats, throughout the years, which were retained in the analyzed bone. Each of these growth layers was also associated with an estimated body size, age, and annual growth reported in the previous skeletochronology analysis of Turner Tomaszewicz, Avens, et al. (2022).

**FIGURE 2:**
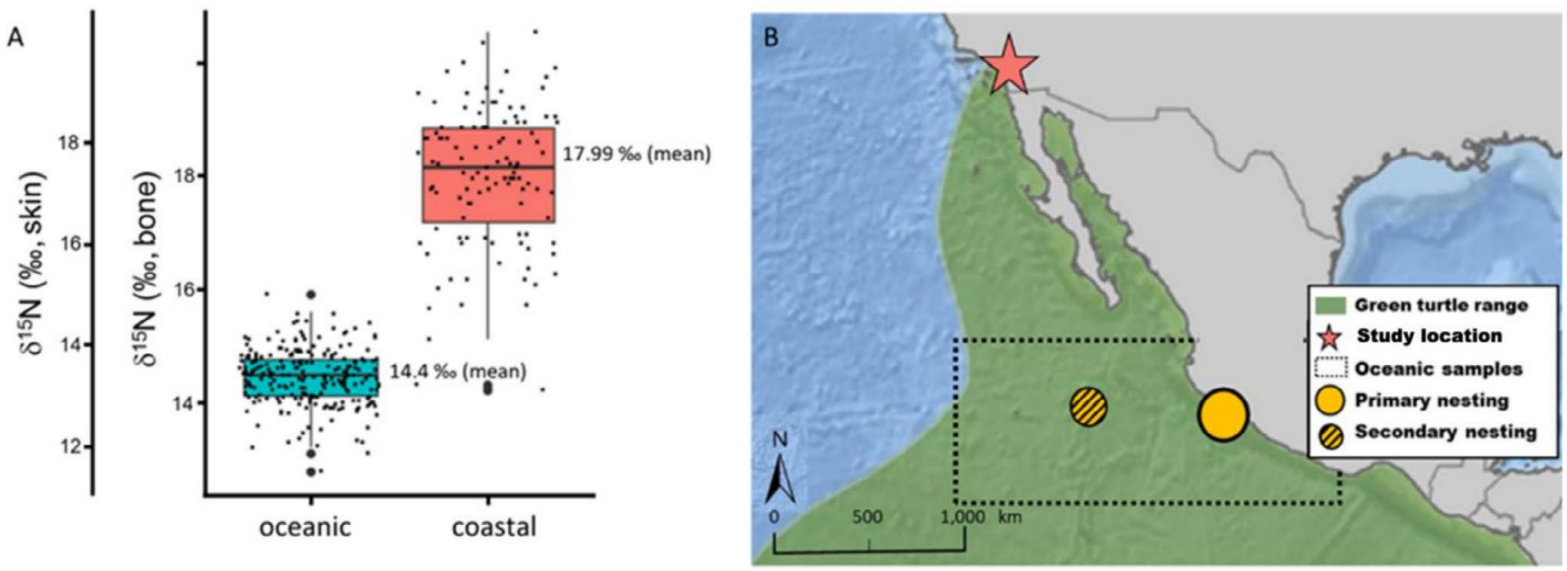
(A) Distribution of neritic (red) and oceanic (blue) skin samples from Seminoff et al. (2021) and Peavey et al. (2017), respectively. Scales are shown for both original skin (left) stable nitrogen isotope values as well as bone‐equivalent values (right), and the mean values provided are also bone‐equivalent (see Methods for details). (B) Map: Green denotes the expected range of EP green sea turtles and the two main northern rookeries at Michoacán (large yellow circle) and Revillagigedo Archipelago (smaller yellow‐black hashed circle); red star shows the study location, also where the coastal samples were collected for Seminoff et al. (2021), and the black dotted line denotes the broad region where oceanic samples were collected for Peavey et al. (2017).

Validating the spatial gradient or pattern in stable isotopes is an important step when inferring habitat use and movement between distinct habitats and/or regions (Vander Zanden et al. forthcoming). For the current study, this was done by first characterizing coastal southern California habitats by using published δ^15^N values of skin from green sea turtles captured and sampled during in‐water research at two southern California locations, San Diego Bay and Seal Beach National Wildlife Refuge in Long Beach (Seminoff et al. 2021) (Figure 2). Ongoing long‐term research projects in these neritic habitats, spanning 30 and 12 years respectively (Seminoff et al. 2021), have shown that green sea turtles are residents in these foraging areas. Seminoff et al. (2021) presented a large sample size (*n* = 112) of skin SI values, with broad spatial and temporal coverage of the two foraging sites, and the data published provided a robust characterization of coastal Southern California green sea turtle foraging habitats.

Then, to isotopically characterize the oceanic habitat of green sea turtles in the eastern North Pacific, we used a different but sympatric species, olive ridleys ( *Lepidochelys olivacea* ), because there are currently no oceanic green sea turtle stable isotope samples from the eastern North Pacific region. We referenced published δ^15^N values from Peavey et al. (2017) of skin from the much more abundant olive ridley sea turtles in oceanic habitats of the eastern North Pacific (Figure 2) where green sea turtles have a known presence (Pitman 1993; Seminoff et al. 2012; NOAA NMFS 2015; Dutton et al. 2019). We felt this was the best and most appropriate proxy to use, as a previously published study has shown that sympatrically foraging green and olive ridley sea turtles in the eastern Pacific have skin δ^15^N and δ^13^C values that are not significantly different (Kelez 2011). Only δ^15^N values of samples from olive ridley turtles captured in the oceanic regions directly offshore from green sea turtle rookeries at Michoacán and the Revillagigedo Archipelago were included, because these are regions where satellite tracking has confirmed the presence of green sea turtles from Southern California foraging areas (Dutton et al. 2019) (Figure 2).

Next, because SI values are tissue‐specific (Vander Zanden et al. forthcoming) and we needed all isotopic values to be directly comparable to bone tissue values, we converted the skin SI values from Seminoff et al. (2021) and Peavey et al. (2017) to bone‐equivalent SI values. For this, we applied the previously published experimentally derived skin‐to‐bone equation from Turner Tomaszewicz, Seminoff, Price, and Kurle (2017) δ^15^N_bone_ = 0.89 (δ^15^N_skin_) + 2.55; as has been fully described and conducted in Turner Tomaszewicz, Seminoff, Price, and Kurle (2017) and Turner Tomaszewicz et al. (2018). The bone‐equivalent δ^15^N values of the selected regions of oceanic olive ridley turtles in Peavey et al. (2017) ranged from 12.8‰ to 15.9‰ (mean ± SD 14.4 ± 0.49) (Table 2), and the neritic green sea turtle skin samples in Seminoff et al. (2021) had bone‐equivalent δ^15^N values that ranged from 14.2‰ to 20.5‰ (mean ± SD 18.0 ± 1.2) (Table 2). The two groups—coastal in Seminoff et al. (2021) and oceanic in Peavey et al. (2017)—are significantly different (Welch two‐sample *t*‐test, *p* < 0.0001, *t* = 29.307, df = 128.55) and the means differed by 3.6‰, a biologically meaningful amount, as trophic levels typically differ by ~3‰ δ^15^N, a magnitude which serves as a good indicator of distinct habitat usage (Minagawa and Wada 1984; Vander Zanden et al. 2005) (Figure 2 and Table 2).

**TABLE 2:** Published data used for habitat characterization, all reported as bone‐equivalent values, converted using the skin‐to‐bone equation from Turner Tomaszewicz, Seminoff, Price, and Kurle (2017).

Study	Region	*n*	δ^15^N mean ± SE	Range	δ^13^C mean ± SE	Range
Peavey et al. 2017 *Bone equivalent*	Oceanic	228	14.4 ± 0.03	12.8–15.9	−16.7 ± 0.01	−17.4 to −16.1
Seminoff et al. 2021 *Bone equivalent*	Neritic	112	18.0 ± 0.12	14.2–20.5	−17.0 ± 0.07	−20.6 to −15.0

Ultimately, this allowed us to characterize the carbon and nitrogen isotopic values in each growth layer, which represented a year of that turtle's life, by matching the bone SI values with the validated isotope values of each habitat region (oceanic vs. coastal). After each growth layer was matched to an isotopic habitat, we evaluated the corresponding demographic data for each layer to align habitat with estimated age, body size, growth, and calendar year as done in Turner Tomaszewicz et al. (2016) and Turner Tomaszewicz, Liles, et al. (2022). To examine any relationship between SI values (dependent variable) and body size and/or age (independent variable), a simple linear regression equation (δ^15^N~CCL or age) was applied in R and *p*‐values and *R* ^2^ values were reported for any significant findings.

To identify the “departure threshold” value, that is, the isotopic threshold indicating when a turtle was likely to have departed the oceanic habitat for a more coastal ecosystem, we referenced the maximum value from the oceanic turtle dataset. The maximum oceanic value was 15.9‰, and therefore, we designated 16‰ δ^15^N as the threshold value (Table 2 and Figure 2). This was 1.6‰ higher than the mean value of all the oceanic samples, and 2‰ lower than the mean value of all of the neritic turtles (18‰), and these differences represent biologically meaningful separation between habitats (Table 2 and Figure 2). Any growth layers with δ^15^N values higher than the 16‰ oceanic departure value would indicate likely settlement to a nearshore foraging site and were assigned to a “coastal” habitat, while growth layers with δ^15^N values lower than 16‰ were assigned to an “oceanic” habitat. Finally, we assigned the age and size at departure from the oceanic habitat, also reflecting the oceanic stage duration, when possible, for each turtle as the first (innermost) growth layer with a δ^15^N value equal to or greater than the 16‰ threshold. Depending on when or if this shift was observed, turtles were classified into one of three groups: shifters, residents, and recent coastal recruits. “Shifters” were those turtles with both oceanic and (then) coastal SI values, “residents” had only coastal SI values, and “recent coastal recruits” had only oceanic SI values but were recovered (dead) in a coastal habitat. Results are presented as mean ± standard error (SE) unless otherwise noted.

### 2.4 Using Multiple Data Sources to Inform Skeleto+Iso Size and Habitat

Finally, we utilized additional data collected during ongoing long‐term research to further inform and interpret the bone demographic and biogeochemical skeleto+iso data, including turtle capture histories and necropsy findings. To inform conclusions about habitat use and to corroborate body sizes (and growth rates) estimated from skeletochronology, we referenced previously collected data from individual turtles when available, such as capture dates, location, morphometric data, body condition, satellite tagging history, and tissue samples collected and analyzed for SI and genetics (Dutton et al. 2019; Seminoff et al. 2021; Maurer et al. 2024; NOAA NMFS, unpublished manuscript). For any turtle that was necropsied, we referenced information such as body condition, indicators of death, stomach contents, and skin SI (NOAA NMFS, unpublished manuscript). The full set of information about individual turtles from multiple data sources was then examined to create the most detailed history possible for all turtles reflecting multi‐year diet and habitat use patterns.

## 3 Results

### 3.1 Stable Isotope Analysis

**FIGURE 3:**
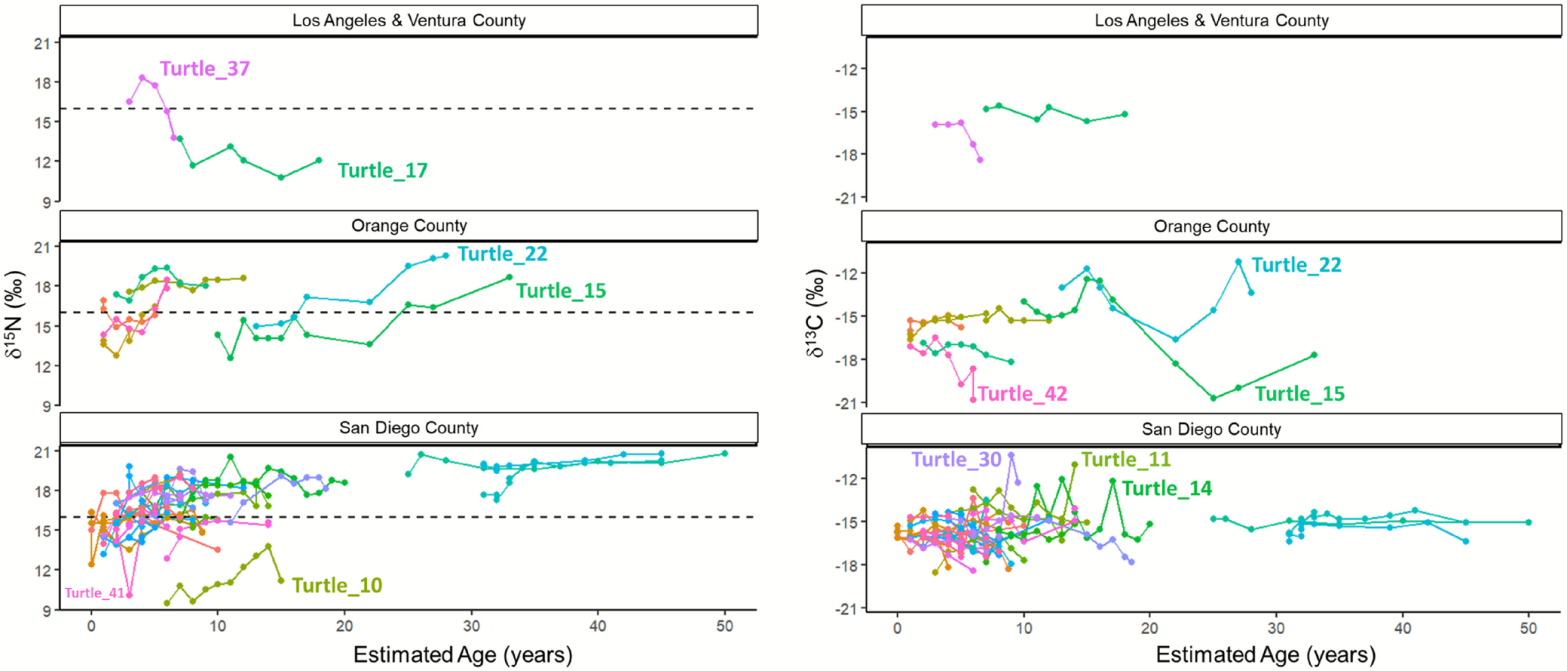
Stable nitrogen (left) and carbon (right) of the 39 turtles grouped by county and aligned to the corresponding estimated age (years). The horizontal dashed line denotes the oceanic departure threshold value of 16‰ δ15N.

**FIGURE 4:**
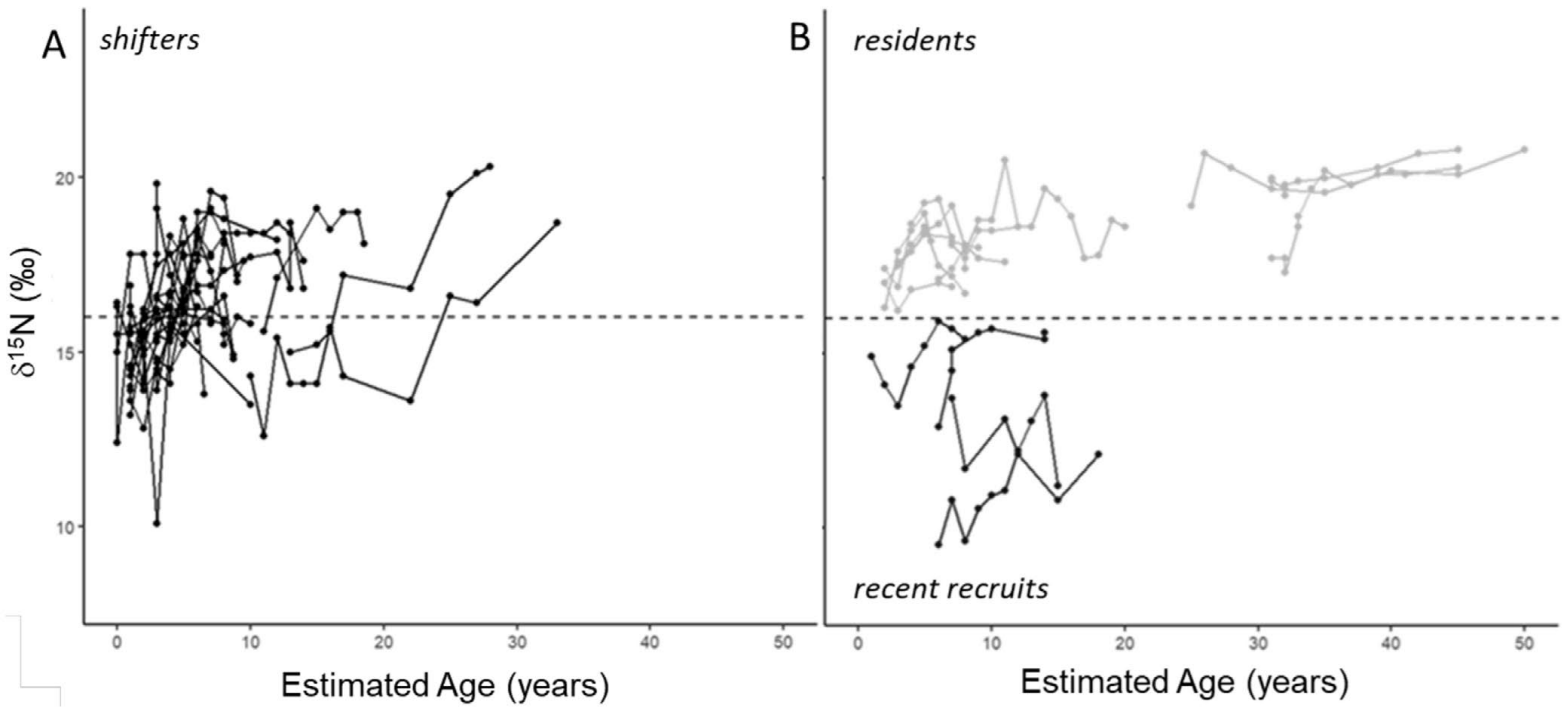
Stable nitrogen isotope values at corresponding estimated age for the turtles based on habitat‐use group. Each line shows an individual turtle, with points showing individual growth layer samples. (A) The 24 “shifter” turtles, (B) the 11 “resident” turtles (gray), and the 4 “recent recruit” turtles (black), as fully described in the Methods. The horizontal dashed line shows the oceanic habitat threshold at 16‰ δ^15^N, above which represents a coastal habitat, and below an oceanic habitat.

A total of 297 samples from growth layers of the 39 turtles' bones were analyzed; the number of growth layers sampled per individual turtle ranged from five to 14. The δ^15^N values ranged from 9.5‰ to 20.8‰ (16.7‰ ± 0.13‰) and the δ^13^C values, all corrected as described in the methods, ranged from −20.8‰ to −10.4‰ (−15.6‰ ± 0.08‰) (Table 3). Turtle recovery sites grouped coarsely into three regions, moving from the northernmost region (Ventura and Los Angeles County) to the central (Orange County) and then the most southern (San Diego County), The mean δ^13^C values were similar, all around −15‰, but with Orange County having the turtle with the lowest (most negative) δ^13^C value (−20.8‰), and with San Diego County having the turtle with the highest (least negative) δ^13^C value (−10.4‰) (Table 3, Figures 1 and 3). In contrast, the mean δ^15^N values varied among the regions, with the lowest mean and (lowest) maximum δ^15^N value in the two turtles recovered in the most northern region (14.1‰ ± 2.6‰; 18.3‰, respectively), and the highest mean and (highest) maximum δ^15^N from turtles recovered in the southern region (16.9‰ ± 2.2‰; 20.8‰, respectively) (Table 3). The central region, Orange County, had the widest range of δ^13^C values, with a maximum value around −12‰, similar to what was also found in San Diego County samples, but with distinct minimum δ^13^C values ca. –21‰ from two different turtles (Turtle 15 and Turtle 42) that were both found in the San Gabriel River (Figures 3 and 4).

**TABLE 3:** Stable nitrogen and carbon values for all bone samples. The stable carbon values are corrected values to account for minimal amounts of inorganic carbon, as fully described in the Methods.

	No. turtles	No. growth layers	δ^15^N mean ± SE	Range	δ^13^C mean ± SE	Range
All	39	297	16.7 ± 0.13	9.5–20.8	−15.6 ± 0.08	−20.8 to −10.4
Los Angeles/Ventura Counties	2	11	14.1 ± 0.77	10.8–18.3	−15.8 ± 0.40	−18.4 to −14.6
Orange County	7	56	16.4 ± 0.27	12.6–20.3	−15.8 ± 0.28	−20.8 to −11.2
San Diego County	30	230	16.9 ± 0.15	9.5–20.8	−15.6 ± 0.08	−18.5 to −10.4

### 3.2 Green Sea Turtle Habitat Assignment and Ontogenetic Shift Timing

Of the 39 turtles, a total of 24 had a mix of both oceanic and coastal δ^15^N values within their bones, and were therefore classified as “shifters” (Figure 4A). For each of these 24, the age and size of the first (innermost) growth layer with a value above the oceanic departure threshold reflected the oceanic stage duration, reported below. The other subsequent growth layers (moving outward) with δ^15^N values equal to or greater than the 16‰ threshold value represented years when the turtles were occupying coastal habitats (Figure 3). A total of 11 turtles were characterized as “resident” turtles, each with all δ^15^N values equal to or greater than the 16‰ threshold (Figure 4B). Finally, four turtles were classified as “recent coastal recruits”, with all δ^15^N values below 16‰ (Figure 4B). For these recent coastal recruits, the size and age at stranding (final) represented their oceanic stage duration, as they all were recovered dead stranded along the coast, and were therefore assumed to have recently departed the oceanic habitat and entered the coastal habitat. Again, we assume these animals had not been foraging in a coastal foodweb for long enough to have incorporated a coastal isotopic signature into their bones, but were occupying a coastal habitat such that they were close enough to shore to have drifted to a beach before sinking or being predated upon, or died and were found within a bay or lagoon.

The age and size at which turtles underwent an ontogenetic habitat shift was estimated for 28 individuals (four recent coastal recruits and 24 shifters), and showed a range of when this shift occurs for green sea turtles in the northern range of the ENP. The estimated oceanic duration ranged from ~one to 25 years (mean: 6.6 ± 1.2 years, median: 5.0 years) with corresponding estimated body size (curved carapace length, CCL) ranging from 23.0 to 96.4 cm (mean: 49.0 ± 3.3 cm, median: 47.8 cm) (Table 4 and Figure 4).

**TABLE 4:** Estimated oceanic duration (years) and corresponding body size (curved carapace length, CCL, cm) at departure from oceanic habitats for the 28 turtles classified as either shifter or recent coastal recruit based on bone stable nitrogen isotope values.

	No. turtles	Estimated CCL at shift (cm)		Estimated age at shift (years)	
		Mean ± SE	Range	Mean ± SE	Range
All	28	49.0 ± 3.3	23.0–96.4	6.6 ± 1.2	~1–25
Los Angeles/Ventura Counties	2	54.2 ± 16.8	37.3–71.0	12.0 ± 9.0	3–21
Orange County	5	64.1 ± 11.3	30.6–96.4	10.0 ± 4.5	1–25
San Diego County	21	45.0 ± 2.9	23.0–78.6	5.2 ± 0.9	~1–15

### 3.3 Multi‐Year Habitat Use and Foraging Patterns

Overall, as turtles grew and aged, their δ^15^N values generally increased. The youngest turtle ages, from 0 to 10 years, had corresponding body sizes up to 65 cm CCL (mean 49 cm, range: 24 to 88.7 cm). During this age and size range, the δ^15^N values increased from ~13‰ up to ~18+‰ (range: 9.5‰ to 19.8‰) and had a significant, albeit weak linear relationship (*p* < 0.001, adj. *R* ^2^ = 0.36) (Figures 3 and 4). The increasing δ^15^N pattern is consistent with the expected pattern of turtles starting in an oceanic habitat, well characterized as depleted in ^15^N relative to neritic coastal habitats, and then settling into more ^15^N‐enriched neritic habitats. Upon settlement into a coastal habitat, all 11 residents, and 16 of the 24 shifters (27 of 39, 69.2%), maintained δ^15^N values above the threshold, suggesting consistent coastal neritic habitat residency (Figures 3 and 4). The three largest and oldest turtles, all residents, showed consistency in their δ^15^N values, with minimal variation over time (ranges for each turtle: 1.3‰, 1.6‰, 3.0‰) across all years sampled (Figures 3 and 4). These results support patterns of site fidelity with long‐term residency and diet specialization for large, older turtles (Figure 3).

Overall, the δ^13^C values did not show any consistent patterns of change, with most individual turtles having values within 1‰ of the overall mean value of −15.6‰ (SE 1.4‰, Table 3, Figures 3 and 4). For individual turtles, the range of δ^13^C values was between 0.6‰ and 8.3‰ (mean 2.5‰). And of the three largest and oldest turtles mentioned above, their δ^13^C values also showed minimal variation (ranges for each turtle: 0.7‰, 1.3‰, 1.6‰), similar to their δ^15^N values. While most turtles had all δ^13^C values around −15‰, there were five turtles with outlier values. Three turtles recovered in the San Diego region had years (i.e., growth layers) with atypically high δ^13^C values (~ −12‰), suggesting higher levels of eelgrass consumption (or foraging in an eelgrass‐based foodweb). Conversely, in the central region of Orange County, two turtles (Turtle 15 and Turtle 42) had noticeably depleted δ^13^C values (~ −20‰), suggesting a unique habitat and diet, different from all the others (Table 3 and Figure 3). Both turtles were found in the San Gabriel River—a highly urbanized waterway with freshwater flow and a great deal of urban and terrestrial input. These outlier δ^13^C values approached ca. –21‰ in some of the turtles' most recent growth layers, recording movement into this isotopically distinct riverine foraging habitat in the last few years of their lives (Figure 3).

### 3.4 Additional Evidence From Live Captures and Necropsies

Of the 39 turtles included in this study and recovered from coastal foraging areas in three different counties (Table 3), eight turtles (20.5%) had been live‐captured previously during in‐water research, with the maximum number of encounters being nine times over a period of nearly 25 years from 1990 to 2014 (Turtle 23). The review of dead‐stranding records from all 39 turtles showed the condition of the carcasses to vary widely, with necropsies conducted on 28 of the 39 (71.8%). Visual inspection of all of the turtles found that nine of the 39 turtles (23.1%) had large barnacles on their carapace and/or plastron, which may indicate some recent time spent in pelagic waters, and these turtles ranged in size from 43.1 to 79.4 cm CCL (mean 50.8 cm).

Of the 28 turtles with necropsy analysis, details about digestive tract contents were available for 23 individuals (82.1%), and were referenced to ground‐truth inferences from stable isotope values. The necropsy reports document a range of diet items recently consumed by these turtles (items observed in esophagus or upper gastrointestinal tract), with the most common items being red algae (*Gracillaria* sp.; observed in 19 of 23 turtles) and then eelgrass ( *Zostera marina* ; 16 of 23). Other items found in smaller amounts and with less frequency included invertebrates such as *Navanax inermis* and other unidentifiable nudibranch species, gastropod egg masses, shells from oysters, clams, snails, and red crustacean (unk sp.), as well as plant matter such as sea lettuce ( *Ulva lactuca* ), kelp (e.g., *Macrocystis* sp.), and filamentous green algae (unk sp).

## 4 Discussion

The general life history pattern of small, young juvenile green sea turtles is well established. They spend time in the oceanic habitat where they can grow before recruiting to coastal habitats (Bolten 2003). In the current study, this movement from an oceanic habitat into a nearshore coastal habitat was recreated using the well‐documented δ^15^N spatial gradient in the ENP by sampling growth layers from green sea turtle bones. In doing so, the biogeochemical patterns within bones allowed for the detection of the timing of this habitat shift of individual turtles, and we were able to estimate the oceanic stage duration for turtles in this population. Stage duration and ontogenetic shift timing are important life history parameters for managers to use in prioritizing conservation efforts, assessing population trends, and making abundance estimates.

Here, we used the isotopic threshold of 16‰ for δ^15^N to identify when 28 green sea turtles made this ontogenetic habitat shift. Based on the age and size corresponding with the bone growth layers where this shift in δ^15^N occurred, this transition appears to typically occur between ages 1 and 10 years (mean age of 6.6 years), and before turtles reach the body size of ~60 cm CCL (mean size of 49.0 cm CCL). Yet the full range of age and size of the 28 turtles also presents evidence that some turtles may remain in the oceanic zone for much longer (over 20 years; Turtle 15 was “oceanic” at an estimated age of 22) (Table 4 and Figure 3). This variation underscores the need to protect and monitor offshore as well as nearshore habitats for green sea turtles. This estimated oceanic stage duration, based on changes in SI values, provides independent and empirical evidence supporting the findings of the previous skeletochronology study (Turner Tomaszewicz, Avens, et al. 2022), which had found the smallest size of turtles documented in near shore habitats during in‐water research efforts of ~50 cm CCL, to correspond to an estimated age, and therefore average oceanic stage duration, of ~5 years (Turner Tomaszewicz, Avens, et al. 2022).

### 4.1 Life History Patterns and Conservation Implications Corroborated Through Multiple Lines of Evidence

Capture‐mark‐recapture research in San Diego Bay over the past several decades has documented long‐term habitat residency of individual turtles (MacDonald et al. 2013; Dutton et al. 2014, 2019; Turner Tomaszewicz, Avens, et al. 2022), and the relatively stable and consistent δ^15^N values from past studies (Lemons et al. 2011; Seminoff et al. 2021), and observed in the current study, show the same pattern of site fidelity. The capture histories—both the dates and the morphometric size data collected—also provided support for the timing of the habitat shift recorded in the bones. Here we review a few case studies presenting information on individual turtles to compare with the conclusions drawn from the skeleto+iso analysis.

**FIGURE 5:**
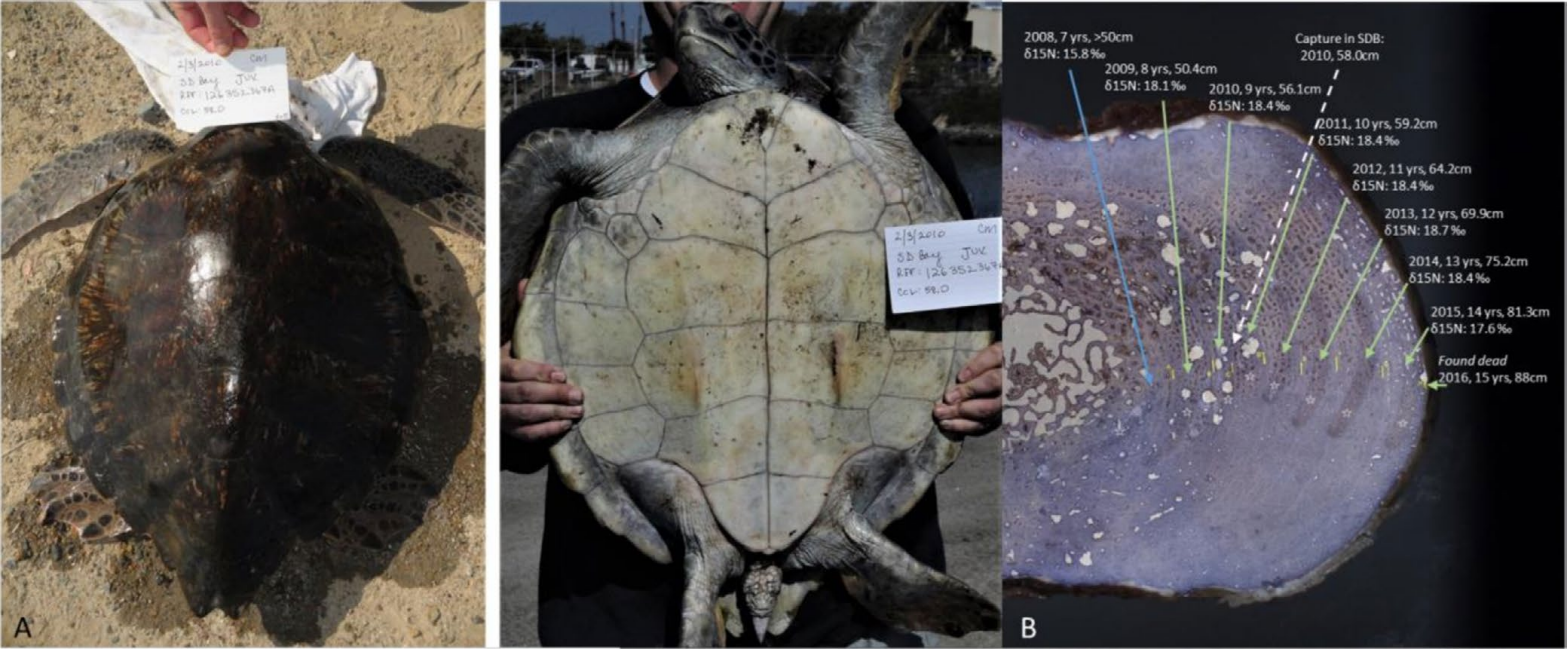
(A) Photos of Turtle 13 at its capture in 2010 in the San Diego Bay. (B) Image of the skeletochronology image with identified growth layers (yellow vertical marks) overlaid upon the stable isotope sampled section (gray stars mark SI sampling lines), with lines identifying each growth layer and associated data: Year, estimated age, estimated body size (CCL), and δ^15^N value. The line color represents the assigned habitat: Oceanic (blue) or coastal (green). The white dashed line shows the estimated timing of the 2010 capture event. The turtle was recovered dead in 2016 (bone outer edge). NOAA Permit #s 1591, 18238, 16803.

First, the bone of Turtle 13 recorded stable δ^15^N (and δ^13^C) values starting in approximately 2008, its only oceanic year having a δ^15^N value of 15.8‰ (< 50 cm CCL, age 7), and in ~2009, the δ^15^N value increased to 18.1‰ indicating recruitment into a nearshore coastal environment at an estimated 50.4 cm CCL, age ~8 (Figure 5). This turtle then appeared to occupy the same isotopic habitat through the remainder of its life, up until it died in 2016 when it was recovered dead‐stranded in San Diego Bay (at ~87 cm CCL and age ~15). All of the SI values from 2009 onward were “coastal”, above 16‰. In 2010, one year after its SI‐presumed habitat shift to the coastal habitat (and 6 years before its death), Turtle 13 was captured during NOAA's in‐water monitoring at the southern end of San Diego Bay. At the time of capture, it had morphological traits consistent with recent recruitment of a small turtle to a neritic habitat—a white plastron and scalloped marginal scutes (Figure 5), and measured 58 cm CCL, which was within the range of the body size estimated by skeletochronology for the corresponding growth layer, 56–59 cm CCL. These physical traits support the timing of recent recruitment to a coastal habitat, as indicated by the SI value for that corresponding growth layer. During its time in San Diego Bay, skeletochronology data indicated that the turtle grew relatively rapidly, with annual growth averaging 5.3 cm (annual growth ranged from 3.1 to 6.2 cm/year), which is consistent with juvenile turtles in this particular habitat (Eguchi et al. 2012; Turner Tomaszewicz, Avens, et al. 2022).

The capture of this turtle and corresponding date and size measurements support key assumptions made when using skeleto+iso: (1) the habitat indicated by the SI value of a particular growth layer, here the years from 2009 onward were assigned as “coastal” and align with capture in San Diego Bay; and (2) the body size estimated using the skeletochronology dimensions of the same growth layer corresponded with the measured CCL during the capture events.

In addition to capturing histories in San Diego Bay supporting the findings from the bone biogeochemical patterns, captures in Long Beach also showed similar support. Turtle 42 was captured alive in 2014 during fieldwork in the very urban habitat of the San Gabriel River, directly adjacent to the SBNWR, a year prior to its death (Figure 1). The bone δ^15^N values indicated departure from the oceanic habitat a year earlier, in 2013 at age ~5 years and ~56 cm CCL. Before this shift to the coastal neritic habitat, the inner bone growth layers recorded 4 years of oceanic habitat use, with δ^15^N values below 16‰ (range 14.3‰–15.5‰), estimated body sizes of 32.8 to 55.6 cm CCL, and from age ~1–5 years. In 2014, when the turtle was caught during in‐water research, the measured body size was 56.6 cm CCL, also within the CCL range estimated by skeletochronology for the 2014 bone growth layer, 56–61 cm CCL. Additionally, the low δ^13^C values (−19.1‰ to −17.3‰) that began in 2013 (the first coastal year) indicate recruitment into and habitat use of the San Gabriel River at that time, and suggest residency in that habitat for ~2 years until its death, where it was recovered just outside the river's mouth along the Bolsa Chica State Beach (at ~7 years old, 63 cm CCL). Again, this habitat has freshwater input and abundant filamentous algae and periphyton, characterized as having lower δ^13^C values in comparison to marine vegetation (Clementz and Koch 2001; Camilleri and Ozersky 2019). As was the case for the turtles captured in San Diego Bay, the recreation of habitat movement using δ^15^N values was corroborated by these in‐water capture events and supports the use of skeleto+iso to recreate movements of green sea turtles in the Southern California region. These data also demonstrate the usefulness of low δ^13^C values to potentially identify when turtles are using the unique San Gabriel River habitat. Future studies, already in progress, will help to validate the reliability of this habitat's isotopic characterization.

Finally, the biogeochemical patterns found in green sea turtle bones revealed additional key insights about the life history patterns of green sea turtles in the ENP, setting the stage for continuing and future studies. Diet items observed and identified during necropsies are found in coastal Southern California habitats, and most have also been previously analyzed for SI analysis in Lemons et al. (2011). As discussed above, SI values from items within coastal habitats provide useful reference values for interpreting turtle SI data; of particular relevance to the current study is the pattern of increasing δ^13^C values with red algae < sea lettuce < eelgrass (Lemons et al. 2011; NOAA NMFS, unpublished manuscript). First, highly regarded as a primary diet item for sea turtles, eelgrass appears to have lower importance in the diet of the green sea turtles in Southern California covered in this study. Only a few turtles had highly enriched δ^13^C values (−12‰ to −10‰) in their bone growth layers that would support the assumption of primarily eelgrass and eelgrass‐based foodweb consumption (Figure 3). Turtle 11 was the one turtle with a very high δ^13^C value in its outermost growth layer, −10.9‰, suggesting recent high eelgrass consumption. In support of this, its necropsy report stated contents as, “Esophagus – 100% *Z. marina* (eelgrass), Upper GI – lots of eelgrass, some red algae”. Different from this turtle, most sea turtles appeared to be consuming a mix of diet items—from both algae and eelgrass foodwebs. This biogeochemical interpretation is supported by examining the items found in the digestive tracts of turtles during necropsies, where 82.6% had red algae, while 69.6% of those necropsied had eelgrass, but percent content estimates were not provided for any turtle other than Turtle 11 remarked on above. The implications of these results are that habitat conservation efforts should focus on the protection of diverse habitats that include a variety of submerged aquatic vegetation, and not just eelgrass beds alone. Further studies examining the degree of eelgrass specialization of sea turtles in Southern California, including studies that utilize newer techniques such as metabarcoding and compound‐specific amino acid fingerprinting, are encouraged to better understand the role of eelgrass and other primary sources in sea turtle habitat ecosystems.

Next, results suggest that some turtles appear to remain in pelagic habitats or move between benthic and pelagic areas, rather than settling fully into neritic bay and lagoon habitats. For example, the small turtle recovered in Los Angeles County (Turtle 37) had earlier (inner) δ^15^N values > 16‰, indicating departure from the oceanic habitat and into a coastal neritic foraging habitat, yet it then appeared to leave the coastal habitat and move back into an oceanic habitat, indicated by the subsequent (outer) δ^15^N values below 16‰ (the outermost growth layer was 13.8‰). This low SI value likely indicates pelagic foraging, even if it was in a coastal zone, and indeed this is further supported by the diet items found in the stomach of this turtle, which included “red crustacean parts” as recorded in the necropsy findings, likely being pelagic red crab (aka tuna crab, *Pleuroncodes planipes* ). Red crabs are a pelagic invertebrate that sea turtles are known to consume in coastal shelf habitats (Peckham et al. 2008, 2011; Turner Tomaszewicz et al. 2018), further supporting the notion of pelagic foraging and habitat use by this turtle. Also, the timing of the recovery of this turtle in 2016 aligns with the well‐documented presence of pelagic red crabs along the US West Coast. This turtle was classified as a “recent recruit” because it was found along a beach, and therefore had died in a coastal region and was in close enough proximity to wash up on shore. Yet we acknowledge that it is very likely that this turtle was not truly recruited to a nearshore neritic habitat, such as a bay or lagoon, but was instead exhibiting an alternative life history strategy with increased use of pelagic habitats for foraging and could have possibly even been in oceanic habitats. This turtle is an example of greens in this population having more flexible habitat movement between oceanic and coastal neritic habitats, and is a pattern that should continue to be explored using additional tools, including satellite telemetry.

We also reintroduce the idea of documenting barnacles and epibionts on turtles, as the presence (and type and location on the turtle's body) could provide evidence of habitat use and/or movement; yet more detailed research focusing on this aspect, particularly for turtles in Southern California is needed. Anecdotally, green sea turtles in San Diego Bay are thought to actively clear barnacles by rubbing on rocks and other submerged objects (see Mullaney et al. 2024), and barnacles found on turtles in this habitat are also observed as becoming packed with sediment, presumably due to the turtle occupying this nearshore habitat which is inhospitable to filter‐feeding epibionts such as turtle barnacles and supports the idea that healthy barnacles could indicate time spent in pelagic, sediment‐free habitats. However, this theory needs further research prior to confirming such conclusions.

A final interesting aspect that should continue to be explored is whether some turtles in Southern California may have occupied or even originated from (rookery/natal beach) the pelagic habitats far offshore, possibly even belonging to the separate Hawaiian population in the remote Central North Pacific (CNP). Two turtles, both recovered in San Diego Bay, had inner δ^15^N values that were much lower (depleted ^15^N) than all the rest of the turtles recovered in Southern California, Turtles 10 and 41 each had values ~10‰. And one turtle, recovered furthest north, Turtle 17, had some low δ^15^N values, including one year at 10.8‰, also suggesting very different habitat use. None of the other 36 turtles sampled in the present study had any values near this low 10‰ δ^15^N value (Figure 3), and the lowest values in the Peavey et al. (2017) study of turtles in the oceanic ENP were 12.8‰ (Table 1). Yet other sea turtle bones from the North Pacific, and analyzed for SI have been found with similarly low δ^15^N values (~10‰). Turner Tomaszewicz, Seminoff, Peckham, et al. (2017) found bones of loggerhead turtles recovered in the CNP had δ^15^N values in this range (mean 10.7‰ ± 1.9‰). A similar pattern was also documented in Allen et al. (2013) which showed loggerhead turtles of the CNP having much lower δ^15^N values than loggerheads from the ENP Baja Peninsula foraging area, by a magnitude of ~5‰. This spatial pattern of δ^15^N values from sea turtle tissues corresponds with other large‐scale isoscape patterns documented for the North Pacific, including those created using data from multiple taxa in Arnoldi et al. (2023) and through ocean nitrogen isotope models in Somes et al. (2010) and Rafter et al. (2019). Given this, it seems likely that green sea turtles in the ENP with bone growth layers with δ^15^N values ~10‰ may indicate at least some habitat use in the more remote pelagic oceanic waters, perhaps in the CNP. Findings from recent genetic studies and applied to samples from the current study support this notion as well.

Dutton et al. (2019) found that the haplotype CmP3.1, which is that of Turtle 41, was most common to the Lalo rookery (French Frigate Shoals) in the Northwestern Hawaiian Archipelago, and is also present, in a smaller percentage, at the Revillagigedo Archipelago in the ENP (Dutton et al. 2019; Horne et al. 2023). Dutton et al. (2019) reported that this haplotype is the only one shared between CNP rookeries (Hawaii) and ENP rookeries. This genetic evidence of the possible origination of Turtle 41 from the Hawaiian Islands supports the idea that low δ^15^N values of other turtles found in the eastern North Pacific could be attributed to time spent in the characteristically low δ^15^N oceanic pelagic waters of the CNP, while simultaneously acknowledging that this turtle (Turtle 41) could also be from the Revillagigedo Archipelago in the ENP. Going forward, additional research combining spatially explicit stable isotope samples with genetic origin information will help to further establish this, and other useful geochemical habitat indicators, further improving our understanding of habitat use and movement of green sea turtles in the North Pacific.

Lastly, we provide notes related to human interactions with these turtles, all found dead in a highly urban coastal region. The necropsies of 28 of the turtles revealed evidence that five of these turtles (17.9%) had signs of interaction with some sort of fishing gear (e.g., fishing line and/or hook found attached to or inside the body), and 22 of the 28 (78.8%) had wounds suggesting a vessel‐strike injury (VSI, e.g., sharp force, propeller wounds, or blunt force injury on the head and/or carapace, Foley et al. 2019); but it is important to note that it is unconfirmed if these injuries were the cause of death or not. Future research and examination are needed to make such conclusions on causes of mortality (Foley et al. 2019) and were beyond the scope of this study, yet underscore the fact that humans and sea turtles both occupy coastal waters and continuing monitoring of sea turtles along the US West Coast should continue for effective marine resource management.

## 5 Conclusion

Here, we use spatial geochemical patterns and stable isotope analysis of turtle bone growth layers to recreate the movements and habitat use patterns of green sea turtles recovered dead along the US West Coast. This work improves the understanding of the habitat use patterns and basic demographic rates for turtles in the ENP. Addressing these data gaps is important for the recovery and management of sea turtles and other marine resources. Specifically, we report estimates of oceanic stage duration, timing of ontogenetic habitat shifts, and multi‐year foraging patterns for eastern Pacific green sea turtles. In this region, green sea turtles depart the oceanic habitat around 6.6 years of age, as indicated by δ^15^N increasing above an informed threshold, but turtles may do so as early as one year old, or may remain in oceanic zones for much longer. This parameter in particular is important for estimating population‐level abundance and forecasting turtle densities in Southern California, essential information in anticipating future management scenarios.

Once settled into a coastal habitat, it appears common for turtles to establish residency, and while some individuals consume seagrass, SI values indicated that it is not the primary diet item of most turtles. By also using live‐capture histories and the findings from necropsies, we were able to corroborate patterns inferred from bone and habitat geochemistry. In addition to bone δ^15^N values > 16‰ indicating departure from the oceanic habitat, other newly identified outlier SI values will also be useful in future studies. This includes much lower bone δ^15^N values (~9‰–11‰) that may suggest time spent in remote oceanic pelagic waters, extremely low δ^13^C values (~ −21‰ to −19‰) likely indicating time in the San Gabriel River habitat, and extremely high δ^13^C values (> −12‰) signifying high levels of eelgrass consumption. These findings fill in important data gaps about green sea turtle demography and habitat use with immediate application to ongoing regional management efforts.

## Author Contributions

**Calandra N. Turner Tomaszewicz:** conceptualization (lead), data curation (lead), formal analysis (lead), investigation (lead), methodology (lead), project administration (lead), writing – original draft (lead). **Erin LaCasella:** resources (supporting), writing – review and editing (supporting). **Garrett E. Lemons:** project administration (supporting), resources (supporting), writing – review and editing (supporting). **Robin LeRoux:** project administration (supporting), resources (supporting), writing – review and editing (supporting). **Jeffrey A. Seminoff:** conceptualization (supporting), formal analysis (supporting), funding acquisition (equal), project administration (equal), resources (equal), supervision (equal), writing – review and editing (equal).

## Funding

The authors have nothing to report.

## Conflicts of Interest

The authors declare no conflicts of interest.

## Supporting information


**Appendix S1:** ece372482‐sup‐0001‐Appendix.docx.

## Data Availability

All the data in the study are provided in the Appendix, and metadata are available at the NOAA InPort repository.
