# Supplementary material for: Green Sea Turtle Recruitment in the Eastern North Pacific: Patterns Identified Using Geochemical Signatures in Bones
Source: Ecol Evol. 2026 Jan 13;16(1):e72482. doi: 10.1002/ece3.72482 (PMC12796844; doi:10.1002/ece3.72482)
Supplement: Supplementary file 1 — Appendix S1: ece372482‐sup‐0001‐Appendix.docx. [file ECE3-16-e72482-s001.docx]

**Appendix**

**Appendix A** - Data provided for each bone growth layer of the 39 turtles, including Turtle ID, identification of partial growth layers, year, estimated CCL (cm), estimated age (yr), estimated annual growth (cm/yr), final CCL (cm), final estimated age (yr), δ^15^N, δ^13^C. **Appendix**

| **Turtle ID** | **Partial growth layer?** | **Year** | **δ^15^N** | **δ^13^C** | **Final CCL (cm)** | **Final Est Age (yr)** | **Estimated Age (yr)** | **Estimated CCL (cm)** | **Estimated annual growth (cm/yr)** |
| --- | --- | --- | --- | --- | --- | --- | --- | --- | --- |
| **01_EPCm** | - | 2007 | 15.0 | -15.78 | 50 | 10 | 0 | NA | NA |
| **01_EPCm** | - | 2008 | 17.8 | -17.1 | 50 | 10 | 1 | NA | NA |
| **01_EPCm** | - | 2009 | 17.8 | -15.78 | 50 | 10 | 2 | 28.5 | NA |
| **01_EPCm** | - | 2010 | 15.5 | -15.78 | 50 | 10 | 3 | 37.4 | 8.9 |
| **01_EPCm** | - | 2011 | 15.8 | -16.02 | 50 | 10 | 4 | 44.8 | 7.4 |
| **01_EPCm** | Y | 2017 | 13.5 | -15.3 | 50 | 10 | 10 | 49.7 | 0 |
| **02_EPCm** | - | 2007 | 16.9 | -16.02 | 49 | 7 | 1 | NA | NA |
| **02_EPCm** | - | 2007 | 16.3 | -15.3 | 49 | 7 | 1 | NA | NA |
| **02_EPCm** | - | 2008 | 14.9 | -15.42 | 49 | 7 | 2 | 31.6 | NA |
| **02_EPCm** | - | 2009 | 15.5 | -15.3 | 49 | 7 | 3 | 38.7 | 7.1 |
| **02_EPCm** | - | 2010 | 15.3 | -15.3 | 49 | 7 | 4 | 41.8 | 3.1 |
| **02_EPCm** | - | 2011 | 15.8 | -15.78 | 49 | 7 | 5 | 45.1 | 3.3 |
| **03_EPCm** | - | 2009 | 16.3 | -16.14 | 51 | 8 | 0 | NA | NA |
| **03_EPCm** | - | 2010 | 15.2 | -16.14 | 51 | 8 | 1 | NA | NA |
| **03_EPCm** | - | 2011 | 14.1 | -16.74 | 51 | 8 | 2 | 26.9 | NA |
| **03_EPCm** | - | 2011 | 15.1 | -16.14 | 51 | 8 | 2 | 26.9 | NA |
| **03_EPCm** | - | 2012 | 16.5 | -16.5 | 51 | 8 | 3 | 33.1 | 6.2 |
| **03_EPCm** | - | 2013 | 16.2 | -16.62 | 51 | 8 | 4 | 39.4 | 6.3 |
| **03_EPCm** | - | 2014 | 15.5 | -15.54 | 51 | 8 | 5 | 42.4 | 3 |
| **03_EPCm** | - | 2017 | 16.6 | -16.98 | 51 | 8 | 8 | 45.6 | 0 |
| **03_EPCm** | Y | 2017 | 14.9 | -18.3 | 51 | 8 | 8.75 | 51 | 5.4 |
| **04_EPCm** | - | 2011 | 16.4 | -15.3 | 43 | 6 | 0 | NA | NA |
| **04_EPCm** | - | 2011 | 12.4 | -16.14 | 43 | 6 | 0 | NA | NA |
| **04_EPCm** | - | 2012 | 16.1 | -16.26 | 43 | 6 | 1 | 24 | NA |
| **04_EPCm** | - | 2012 | 15.6 | -15.9 | 43 | 6 | 1 | 24 | NA |
| **04_EPCm** | - | 2013 | 15.5 | -15.66 | 43 | 6 | 2 | 25.7 | 1.7 |
| **04_EPCm** | - | 2014 | 15.9 | -15.78 | 43 | 6 | 3 | 31.8 | 6.1 |
| **04_EPCm** | - | 2015 | 16.6 | -18.18 | 43 | 6 | 4 | 39.5 | 7.7 |
| **05_EPCm** | - | 2009 | 15.5 | -15.66 | 53 | 8 | 0 | NA | NA |
| **05_EPCm** | - | 2010 | 15.5 | -15.66 | 53 | 8 | 1 | 28.6 | NA |
| **05_EPCm** | - | 2010 | 15.7 | -16.02 | 53 | 8 | 1 | 28.6 | NA |
| **05_EPCm** | - | 2011 | 15.9 | -15.78 | 53 | 8 | 2 | 32.8 | 4.2 |
| **05_EPCm** | - | 2012 | 16.1 | -15.18 | 53 | 8 | 3 | 36.2 | 3.4 |
| **05_EPCm** | - | 2013 | 16.2 | -15.54 | 53 | 8 | 4 | 40.2 | 4 |
| **05_EPCm** | - | 2016 | 16.2 | -14.82 | 53 | 8 | 7 | 46.2 | 1.1 |
| **05_EPCm** | - | 2017 | 15.9 | -14.34 | 53 | 8 | 8 | 48.1 | 1.9 |
| **05_EPCm** | Y | 2017 | 14.8 | -15.06 | 53 | 8 | 8.75 | 53 | 4.9 |
| **06_EPCm** | - | 2010 | 14.9 | -14.94 | 49 | 8 | 1 | NA | NA |
| **06_EPCm** | - | 2011 | 14.1 | -14.22 | 49 | 8 | 2 | 29.5 | NA |
| **06_EPCm** | - | 2012 | 13.5 | -15.42 | 49 | 8 | 3 | 32.3 | 2.8 |
| **06_EPCm** | - | 2013 | 14.6 | -15.9 | 49 | 8 | 4 | 37.1 | 4.8 |
| **06_EPCm** | - | 2014 | 15.2 | -14.94 | 49 | 8 | 5 | 40.4 | 3.3 |
| **06_EPCm** | - | 2015 | 15.9 | -14.94 | 49 | 8 | 6 | 44.3 | 3.9 |
| **06_EPCm** | - | 2016 | 15.7 | -15.18 | 49 | 8 | 7 | 47.4 | 3.1 |
| **06_EPCm** | - | 2017 | 15.4 | -15.54 | 49 | 8 | 8 | 48.5 | 1.1 |
| **07_EPCm** | - | 2009 | 13.9 | -16.26 | 60 | 6 | 1 | NA | NA |
| **07_EPCm** | - | 2009 | 13.6 | -16.62 | 60 | 6 | 1 | NA | NA |
| **07_EPCm** | - | 2010 | 12.8 | -15.54 | 60 | 6 | 2 | 36.2 | NA |
| **07_EPCm** | - | 2011 | 14.7 | -15.18 | 60 | 6 | 3 | 45.7 | 9.5 |
| **07_EPCm** | - | 2011 | 13.9 | -15.18 | 60 | 6 | 3 | 45.7 | 9.5 |
| **07_EPCm** | - | 2012 | 15.8 | -14.94 | 60 | 6 | 4 | 53 | 7.3 |
| **07_EPCm** | - | 2013 | 16.5 | -15.06 | 60 | 6 | 5 | 57.3 | 4.3 |
| **08_EPCm** | - | 2009 | 17.5 | -18.54 | 66 | 9 | 3 | NA | NA |
| **08_EPCm** | - | 2010 | 17.9 | -17.1 | 66 | 9 | 4 | 42.2 | NA |
| **08_EPCm** | - | 2011 | 18.5 | -16.98 | 66 | 9 | 5 | 48.9 | 6.7 |
| **08_EPCm** | - | 2012 | 18.7 | -16.14 | 66 | 9 | 6 | 56.9 | 8 |
| **08_EPCm** | - | 2013 | 19.2 | -16.14 | 66 | 9 | 7 | 62.1 | 5.2 |
| **08_EPCm** | - | 2014 | 18 | -16.74 | 66 | 9 | 8 | 65.5 | 3.4 |
| **09_EPCm** | - | 2004 | 17.6 | -15.3 | 71 | 13 | 3 | 44.5 | NA |
| **09_EPCm** | - | 2005 | 17.9 | -15.3 | 71 | 13 | 4 | 50.1 | 5.6 |
| **09_EPCm** | - | 2006 | 18.4 | -15.06 | 71 | 13 | 5 | 55.3 | 5.2 |
| **09_EPCm** | - | 2008 | 18.3 | -14.82 | 71 | 13 | 7 | 60.2 | 4.6 |
| **09_EPCm** | - | 2008 | 18.1 | -15.3 | 71 | 13 | 7 | 60.2 | 4.6 |
| **09_EPCm** | - | 2009 | 17.7 | -14.46 | 71 | 13 | 8 | 61.9 | 1.7 |
| **09_EPCm** | - | 2010 | 18.5 | -15.3 | 71 | 13 | 9 | 64 | 2.1 |
| **09_EPCm** | - | 2011 | 18.5 | -15.3 | 71 | 13 | 10 | 65.7 | 1.7 |
| **09_EPCm** | - | 2013 | 18.6 | -15.3 | 71 | 13 | 12 | 69.8 | 2.3 |
| **10_EPCm** | - | 2006 | 9.53 | -12.78 | 79 | 16 | 6 | 49.9 | NA |
| **10_EPCm** | - | 2007 | 10.79 | -13.84 | 79 | 16 | 7 | 51 | 1.1 |
| **10_EPCm** | - | 2008 | 9.64 | -12.84 | 79 | 16 | 8 | 54.7 | 3.7 |
| **10_EPCm** | - | 2009 | 10.55 | -14.03 | 79 | 16 | 9 | 57.9 | 3.2 |
| **10_EPCm** | - | 2010 | 10.94 | -14.81 | 79 | 16 | 10 | 62.2 | 4.3 |
| **10_EPCm** | - | 2011 | 11.06 | -13.67 | 79 | 16 | 11 | 64.7 | 2.5 |
| **10_EPCm** | - | 2012 | 12.22 | -14.54 | 79 | 16 | 12 | 67.9 | 3.2 |
| **10_EPCm** | - | 2013 | 13.06 | -14.83 | 79 | 16 | 13 | 72 | 4.1 |
| **10_EPCm** | - | 2014 | 13.79 | -15.06 | 79 | 16 | 14 | 75 | 3 |
| **10_EPCm** | - | 2015 | 11.2 | -15.06 | 79 | 16 | 15 | 78.6 | 3.6 |
| **11_EPCm** | - | 2007 | 16 | -14.34 | 77 | 14 | 4 | NA | NA |
| **11_EPCm** | - | 2008 | 16.48 | -14.21 | 77 | 14 | 5 | 47.1 | NA |
| **11_EPCm** | - | 2009 | 16.9 | -14.09 | 77 | 14 | 6 | 53.7 | 6.6 |
| **11_EPCm** | - | 2010 | 16.9 | -13.62 | 77 | 14 | 7 | 57.5 | 3.8 |
| **11_EPCm** | - | 2011 | 17.32 | -14.33 | 77 | 14 | 8 | 61.1 | 3.6 |
| **11_EPCm** | - | 2013 | 17.72 | -14.92 | 77 | 14 | 10 | 64.2 | 0.5 |
| **11_EPCm** | - | 2015 | 17.84 | -14.8 | 77 | 14 | 12 | 65.5 | 0.2 |
| **11_EPCm** | - | 2016 | 16.82 | -16.37 | 77 | 14 | 13 | 66.3 | 0.8 |
| **11_EPCm** | - | 2016 | 18.68 | -15.05 | 77 | 14 | 13 | 66.3 | 0.8 |
| **11_EPCm** | - | 2017 | 16.81 | -11.03 | 77 | 14 | 14 | 73.3 | 7 |
| **12_EPCm** | - | 2010 | 17.8 | -16.02 | 75 | 10 | 4 | 43.4 | NA |
| **12_EPCm** | - | 2011 | 16.7 | -16.5 | 75 | 10 | 5 | 50.9 | 7.5 |
| **12_EPCm** | - | 2012 | 18.3 | -16.26 | 75 | 10 | 6 | 58.1 | 7.2 |
| **12_EPCm** | - | 2013 | 17.3 | -16.74 | 75 | 10 | 7 | 60 | 1.9 |
| **12_EPCm** | - | 2014 | 15.5 | -16.26 | 75 | 10 | 8 | 67.6 | 7.6 |
| **12_EPCm** | - | 2014 | 15.2 | -15.78 | 75 | 10 | 8 | 67.6 | 7.6 |
| **12_EPCm** | - | 2015 | 16 | -16.86 | 75 | 10 | 9 | 71.8 | 4.2 |
| **12_EPCm** | - | 2016 | 15.8 | -17.7 | 75 | 10 | 10 | 74.2 | 2.4 |
| **13_EPCm** | - | 2008 | 15.8 | -17.82 | 88 | 15 | 7 | NA | NA |
| **13_EPCm** | - | 2009 | 18.1 | -15.54 | 88 | 15 | 8 | 50.4 | NA |
| **13_EPCm** | - | 2009 | 18.4 | -16.26 | 88 | 15 | 8 | 50.4 | NA |
| **13_EPCm** | - | 2010 | 18.4 | -16.02 | 88 | 15 | 9 | 56.1 | 5.7 |
| **13_EPCm** | - | 2011 | 18.4 | -15.9 | 88 | 15 | 10 | 59.2 | 3.1 |
| **13_EPCm** | - | 2012 | 18.4 | -15.66 | 88 | 15 | 11 | 64.2 | 5 |
| **13_EPCm** | - | 2013 | 18.7 | -16.26 | 88 | 15 | 12 | 69.9 | 5.7 |
| **13_EPCm** | - | 2014 | 18.4 | -15.9 | 88 | 15 | 13 | 75.2 | 5.3 |
| **13_EPCm** | - | 2015 | 17.6 | -15.18 | 88 | 15 | 14 | 81.3 | 6.1 |
| **14_EPCm** | - | 2005 | 17.4 | -16.26 | 87 | 20 | 8 | 49.6 | NA |
| **14_EPCm** | - | 2005 | 17.9 | -15.66 | 87 | 20 | 8 | 49.6 | 0 |
| **14_EPCm** | - | 2006 | 18.8 | -15.9 | 87 | 20 | 9 | 51.8 | 2.2 |
| **14_EPCm** | - | 2007 | 18.8 | -16.26 | 87 | 20 | 10 | 54.2 | 2.4 |
| **14_EPCm** | - | 2008 | 20.5 | -12.54 | 87 | 20 | 11 | 55.9 | 1.7 |
| **14_EPCm** | - | 2009 | 18.6 | -14.7 | 87 | 20 | 12 | 57.6 | 1.7 |
| **14_EPCm** | - | 2010 | 18.6 | -12.06 | 87 | 20 | 13 | 61.3 | 3.7 |
| **14_EPCm** | - | 2011 | 19.7 | -14.34 | 87 | 20 | 14 | 66.5 | 5.2 |
| **14_EPCm** | - | 2012 | 19.4 | -16.14 | 87 | 20 | 15 | 70.3 | 3.8 |
| **14_EPCm** | - | 2013 | 18.9 | -15.54 | 87 | 20 | 16 | 73.3 | 3 |
| **14_EPCm** | - | 2014 | 17.7 | -12.18 | 87 | 20 | 17 | 75.5 | 2.2 |
| **14_EPCm** | - | 2015 | 17.8 | -15.9 | 87 | 20 | 18 | 79.8 | 4.3 |
| **14_EPCm** | - | 2016 | 18.8 | -16.26 | 87 | 20 | 19 | 84.4 | 4.6 |
| **14_EPCm** | - | 2017 | 18.6 | -15.18 | 87 | 20 | 20 | 86.9 | 2.5 |
| **15_EPCm** | - | 1993 | 14.3 | -13.98 | 99 | 33 | 10 | NA | NA |
| **15_EPCm** | - | 1994 | 12.6 | -14.7 | 99 | 33 | 11 | 64.2 | NA |
| **15_EPCm** | - | 1995 | 15.4 | -15.06 | 99 | 33 | 12 | 72.3 | 8.1 |
| **15_EPCm** | - | 1996 | 14.1 | -14.94 | 99 | 33 | 13 | 78.4 | 6.1 |
| **15_EPCm** | - | 1997 | 14.1 | -14.58 | 99 | 33 | 14 | 83.2 | 4.8 |
| **15_EPCm** | - | 1998 | 14.1 | -12.42 | 99 | 33 | 15 | 86.2 | 3 |
| **15_EPCm** | - | 1999 | 15.7 | -12.54 | 99 | 33 | 16 | 89.3 | 3.1 |
| **15_EPCm** | - | 2000 | 14.3 | -13.86 | 99 | 33 | 17 | 91 | 1.7 |
| **15_EPCm** | - | 2005 | 13.6 | -18.3 | 99 | 33 | 22 | 94.7 | 0 |
| **15_EPCm** | - | 2008 | 16.6 | -20.7 | 99 | 33 | 25 | 96.4 | 0.5 |
| **15_EPCm** | - | 2010 | 16.4 | -19.98 | 99 | 33 | 27 | 97 | 0.6 |
| **15_EPCm** | - | 2016 | 18.7 | -17.7 | 99 | 33 | 33 | 98.1 | 0 |
| **17_EPCm** | - | 2001 | 13.7 | -14.82 | 71 | 21 | 7 | NA | NA |
| **17_EPCm** | - | 2002 | 11.7 | -14.58 | 71 | 21 | 8 | 51.9 | NA |
| **17_EPCm** | - | 2005 | 13.1 | -15.54 | 71 | 21 | 11 | 62.1 | 0 |
| **17_EPCm** | - | 2006 | 12.1 | -14.7 | 71 | 21 | 12 | 62.3 | 0.2 |
| **17_EPCm** | - | 2009 | 10.8 | -15.66 | 71 | 21 | 15 | 68.4 | 1.6 |
| **17_EPCm** | - | 2012 | 12.1 | -15.18 | 71 | 21 | 18 | 68.9 | 0.3 |
| **18_EPCm** | - | 2008 | 17.4 | -16.86 | 72 | 10 | 2 | NA | NA |
| **18_EPCm** | - | 2009 | 16.9 | -17.58 | 72 | 10 | 3 | 41.9 | NA |
| **18_EPCm** | - | 2010 | 18.7 | -16.98 | 72 | 10 | 4 | 49.1 | 7.2 |
| **18_EPCm** | - | 2011 | 19.3 | -16.98 | 72 | 10 | 5 | 56.6 | 7.5 |
| **18_EPCm** | - | 2012 | 19.4 | -17.1 | 72 | 10 | 6 | 59.8 | 3.2 |
| **18_EPCm** | - | 2013 | 18.2 | -17.7 | 72 | 10 | 7 | 63.6 | 3.8 |
| **18_EPCm** | - | 2015 | 18 | -18.18 | 72 | 10 | 9 | 69.4 | 0.9 |
| **19_EPCm** | - | 1984 | 19.2 | -14.82 | 96 | 50 | 25 | 90.8 | 0.3 |
| **19_EPCm** | - | 1985 | 20.7 | -14.82 | 96 | 50 | 26 | 91.1 | 0.3 |
| **19_EPCm** | - | 1987 | 20.3 | -15.54 | 96 | 50 | 28 | 91.9 | 0.2 |
| **19_EPCm** | - | 1990 | 19.7 | -14.94 | 96 | 50 | 31 | 92.8 | 0.3 |
| **19_EPCm** | - | 1994 | 19.6 | -15.18 | 96 | 50 | 35 | 93.6 | 0 |
| **19_EPCm** | - | 1999 | 20.2 | -14.94 | 96 | 50 | 40 | 94.2 | 0 |
| **19_EPCm** | - | 2004 | 20.1 | -15.06 | 96 | 50 | 45 | 94.8 | 0 |
| **19_EPCm** | - | 2009 | 20.8 | -15.06 | 96 | 50 | 50 | 95.9 | 0.3 |
| **20_EPCm** | - | 2011 | 17 | -15.9 | 63 | 7 | 2 | NA | NA |
| **20_EPCm** | - | 2012 | 16.2 | -16.14 | 63 | 7 | 3 | 37 | NA |
| **20_EPCm** | - | 2013 | 16.8 | -16.02 | 63 | 7 | 4 | 47.8 | 10.8 |
| **20_EPCm** | - | 2015 | 17 | -16.86 | 63 | 7 | 6 | 59.7 | 0.6 |
| **20_EPCm** | - | 2016 | 16.9 | -13.5 | 63 | 7 | 7 | 60.7 | 1 |
| **21_EPCm** | - | 1999 | 17.7 | -15.78 | 109 | 48 | 31 | NA | NA |
| **21_EPCm** | - | 2000 | 17.7 | -14.82 | 109 | 48 | 32 | NA | NA |
| **21_EPCm** | - | 2001 | 18.6 | -14.34 | 109 | 48 | 33 | NA | NA |
| **21_EPCm** | - | 2002 | 19.7 | -14.46 | 109 | 48 | 34 | NA | NA |
| **21_EPCm** | - | 2003 | 20.2 | -14.82 | 109 | 48 | 35 | 100.1 | NA |
| **21_EPCm** | - | 2005 | 19.8 | -14.82 | 109 | 48 | 37 | 105.1 | 1.4 |
| **21_EPCm** | - | 2007 | 20.1 | -14.58 | 109 | 48 | 39 | 107 | 1.1 |
| **21_EPCm** | - | 2009 | 20.1 | -14.22 | 109 | 48 | 41 | 107.5 | 0.2 |
| **21_EPCm** | - | 2013 | 20.3 | -15.06 | 109 | 48 | 45 | 108.6 | 0.5 |
| **21_EPCm** | - | NA | 17.3 | -15.06 | 109 | 48 | 32 | NA | NA |
| **21_EPCm** | - | NA | 18.9 | -14.7 | 109 | 48 | 33 | NA | NA |
| **21_EPCm** | - | NA | 19.8 | -14.46 | 109 | 48 | NA | 100 | NA |
| **21_EPCm** | - | NA | 19.9 | -14.46 | 109 | 48 | NA | 100 | NA |
| **21_EPCm** | - | NA | 19.7 | -14.58 | 109 | 48 | NA | NA | NA |
| **22_EPCm** | - | 1994 | 15 | -13.02 | 93 | 32 | 13 | 68.7 | 6.9 |
| **22_EPCm** | - | 1996 | 15.2 | -11.7 | 93 | 32 | 15 | 73.7 | 4.4 |
| **22_EPCm** | - | 1997 | 15.6 | -13.02 | 93 | 32 | 16 | 79.2 | 5.5 |
| **22_EPCm** | - | 1998 | 17.2 | -14.46 | 93 | 32 | 17 | 80.4 | 1.2 |
| **22_EPCm** | - | 2003 | 16.8 | -16.62 | 93 | 32 | 22 | 85.4 | 3.1 |
| **22_EPCm** | - | 2006 | 19.5 | -14.58 | 93 | 32 | 25 | 89.6 | 1.7 |
| **22_EPCm** | - | 2008 | 20.1 | -11.22 | 93 | 32 | 27 | 91.6 | 0.9 |
| **22_EPCm** | - | 2009 | 20.3 | -13.38 | 93 | 32 | 28 | 92.2 | 0.6 |
| **23_EPCm** | - | 1999 | 20 | -16.38 | 110.5 | 46 | 31 | 107.5 | 0.3 |
| **23_EPCm** | - | 1999 | 19.9 | -15.9 | 110.5 | 46 | 31 | 107.5 | 0.3 |
| **23_EPCm** | - | 2000 | 19.7 | -16.02 | 110.5 | 46 | 32 | 107.8 | 0.3 |
| **23_EPCm** | - | 2000 | 19.5 | -15.54 | 110.5 | 46 | 32 | 107.8 | 0.3 |
| **23_EPCm** | - | 2000 | 19.8 | -15.18 | 110.5 | 46 | 32 | 107.8 | 0.3 |
| **23_EPCm** | - | 2001 | 19.9 | -15.18 | 110.5 | 46 | 33 | 108 | 0.2 |
| **23_EPCm** | - | 2003 | 20 | -15.3 | 110.5 | 46 | 35 | 108.7 | 0.3 |
| **23_EPCm** | - | 2007 | 20.3 | -15.42 | 110.5 | 46 | 39 | 110.2 | 0.4 |
| **23_EPCm** | - | 2010 | 20.7 | -15.06 | 110.5 | 46 | 42 | 110.7 | -0.1 |
| **23_EPCm** | - | 2013 | 20.8 | -16.38 | 110.5 | 46 | 45 | 110.7 | 0.3 |
| **24_EPCm** | - | 2007 | 15.6 | -16.14 | 54 | 9 | 2 | 31 | NA |
| **24_EPCm** | - | 2008 | 16.2 | -16.14 | 54 | 9 | 3 | 33.4 | 2.4 |
| **24_EPCm** | - | 2009 | 15.3 | -15.66 | 54 | 9 | 4 | 37.2 | 3.8 |
| **24_EPCm** | - | 2011 | 18.4 | -17.1 | 54 | 9 | 6 | 42.4 | 4.9 |
| **24_EPCm** | - | 2012 | 17.8 | -17.22 | 54 | 9 | 7 | 47 | 4.6 |
| **24_EPCm** | - | 2013 | 18.4 | -16.74 | 54 | 9 | 8 | 51.8 | 4.8 |
| **24_EPCm** | - | 2014 | 17 | -17.94 | 54 | 9 | 9 | 53 | 1.2 |
| **25_EPCm** | - | 2009 | 14.5 | -15.3 | 56 | 6 | 1 | NA | NA |
| **25_EPCm** | - | 2010 | 13.9 | -14.94 | 56 | 6 | 2 | 33.9 | NA |
| **25_EPCm** | - | 2011 | 14.5 | -14.82 | 56 | 6 | 3 | 38 | 4.1 |
| **25_EPCm** | - | 2011 | 15.7 | -14.94 | 56 | 6 | 4 | 49.5 | 11.5 |
| **25_EPCm** | - | 2012 | 15.2 | -15.42 | 56 | 6 | 5 | 52.2 | 2.7 |
| **25_EPCm** | - | 2013 | 16.3 | -15.18 | 56 | 6 | 6 | 54.6 | 2.4 |
| **26_EPCm** | - | 2009 | 13.2 | -16.14 | 53 | 8 | 1 | 26.4 | NA |
| **26_EPCm** | - | 2010 | 15.4 | -15.9 | 53 | 8 | 2 | 29 | 2.6 |
| **26_EPCm** | - | 2011 | 17.8 | -16.14 | 53 | 8 | 3 | 38 | 9 |
| **26_EPCm** | - | 2011 | 19.8 | -16.14 | 53 | 8 | 3 | 38 | 9 |
| **26_EPCm** | - | 2011 | 19.1 | -16.02 | 53 | 8 | 3 | 38 | 9 |
| **26_EPCm** | - | 2012 | 17.2 | -16.26 | 53 | 8 | 4 | 45.3 | 7.3 |
| **26_EPCm** | - | 2013 | 16.1 | -15.66 | 53 | 8 | 5 | 48.2 | 2.9 |
| **26_EPCm** | - | 2016 | 15.8 | -17.34 | 53 | 8 | 8 | 52.2 | 1.6 |
| **27_EPCm** | - | 2007 | 14.4 | -14.46 | 58 | 12 | 3 | 34.3 | NA |
| **27_EPCm** | - | 2008 | 14.1 | -14.7 | 58 | 12 | 4 | 37.4 | 3.1 |
| **27_EPCm** | - | 2008 | 14.5 | -14.34 | 58 | 12 | 4 | 37.4 | 3.1 |
| **27_EPCm** | - | 2009 | 15.2 | -14.46 | 58 | 12 | 5 | 41.5 | 4.1 |
| **27_EPCm** | - | 2009 | 15.2 | -14.58 | 58 | 12 | 5 | 41.5 | 4.1 |
| **27_EPCm** | - | 2010 | 19 | -15.42 | 58 | 12 | 6 | 45.7 | 4.2 |
| **27_EPCm** | - | 2011 | 19 | -16.02 | 58 | 12 | 7 | 49.2 | 3.5 |
| **27_EPCm** | - | 2012 | 18.8 | -16.38 | 58 | 12 | 8 | 53.9 | 4.7 |
| **27_EPCm** | - | 2016 | 18.2 | -14.82 | 58 | 12 | 12 | 57.9 | 2.3 |
| **28_EPCm** | - | 2007 | 17 | -16.74 | 86 | 9 | 2 | 41.7 | NA |
| **28_EPCm** | - | 2008 | 17.5 | -15.78 | 86 | 9 | 3 | 49.2 | 7.5 |
| **28_EPCm** | - | 2009 | 18.1 | -15.54 | 86 | 9 | 4 | 59.8 | 10.6 |
| **28_EPCm** | - | 2010 | 18.6 | -15.9 | 86 | 9 | 5 | 67.8 | 8 |
| **28_EPCm** | - | 2011 | 17.5 | -15.66 | 86 | 9 | 6 | 78.3 | 10.5 |
| **28_EPCm** | - | 2012 | 17.2 | -16.5 | 86 | 9 | 7 | 81.4 | 3.1 |
| **28_EPCm** | - | 2013 | 16.7 | -16.14 | 86 | 9 | 8 | 84.9 | 3.5 |
| **29_EPCm** | - | 2008 | 15.6 | -14.82 | 80 | 18 | 11 | NA | NA |
| **29_EPCm** | - | 2009 | 17.1 | -14.94 | 80 | 18 | 12 | 61.2 | NA |
| **29_EPCm** | - | 2012 | 19.1 | -15.9 | 80 | 18 | 15 | 64.1 | 2.1 |
| **29_EPCm** | - | 2013 | 18.5 | -16.74 | 80 | 18 | 16 | 67.4 | 3.3 |
| **29_EPCm** | - | 2014 | 19 | -16.26 | 80 | 18 | 17 | 72.6 | 5.2 |
| **29_EPCm** | - | 2015 | 19 | -17.46 | 80 | 18 | 18 | 75.5 | 2.9 |
| **29_EPCm** | Y | 2015 | 18.1 | -17.82 | 80 | 18 | 18.5 | 80 | 4.5 |
| **30_EPCm** | - | 2009 | 15.8 | -14.7 | 89 | 9 | 4 | 45 | NA |
| **30_EPCm** | - | 2010 | 16.2 | -14.94 | 89 | 9 | 5 | 53.7 | 8.7 |
| **30_EPCm** | - | 2011 | 17.6 | -16.02 | 89 | 9 | 6 | 59.5 | 5.8 |
| **30_EPCm** | - | 2012 | 19.6 | -17.22 | 89 | 9 | 7 | 68.4 | 8.9 |
| **30_EPCm** | - | 2013 | 19.4 | -16.02 | 89 | 9 | 8 | 74.8 | 6.4 |
| **30_EPCm** | - | 2014 | 17.2 | -10.38 | 89 | 9 | 9 | 82.7 | 7.9 |
| **30_EPCm** | Y | 2015 | 17.6 | -12.3 | 89 | 9 | 9.5 | 88.7 | 6 |
| **31_EPCm** | - | 2009 | 17.1 | -15.78 | 67 | 13 | 6 | 55.3 | 8.5 |
| **31_EPCm** | - | 2010 | 17.4 | -15.54 | 67 | 13 | 7 | 61.8 | 6.5 |
| **31_EPCm** | - | 2011 | 18.1 | -14.94 | 67 | 13 | 8 | 63.1 | 1.3 |
| **31_EPCm** | - | 2012 | 17.7 | -14.58 | 67 | 13 | 9 | 63.6 | 0.5 |
| **31_EPCm** | - | 2014 | 17.6 | -14.7 | 67 | 13 | 11 | 66.5 | 0.3 |
| **32_EPCm** | - | 2008 | 14.6 | -16.26 | 65 | 8 | 1 | 31.3 | NA |
| **32_EPCm** | - | 2009 | 16.2 | -16.86 | 65 | 8 | 2 | 38.9 | 7.6 |
| **32_EPCm** | - | 2010 | 17.5 | -16.38 | 65 | 8 | 3 | 44.7 | 5.8 |
| **32_EPCm** | - | 2011 | 17.8 | -16.38 | 65 | 8 | 4 | 50 | 5.3 |
| **32_EPCm** | - | 2012 | 18.1 | -16.38 | 65 | 8 | 5 | 56.8 | 6.8 |
| **32_EPCm** | - | 2012 | 17.8 | -16.86 | 65 | 8 | 5 | 56.8 | 6.8 |
| **32_EPCm** | - | 2014 | 17.7 | -16.86 | 65 | 8 | 7 | 63.4 | 3.8 |
| **37_EPCm** | - | 2012 | 16.5 | -15.9 | 54 | 6 | 3 | 37.3 | NA |
| **37_EPCm** | - | 2013 | 18.3 | -15.9 | 54 | 6 | 4 | 47.8 | 10.5 |
| **37_EPCm** | - | 2014 | 17.7 | -15.78 | 54 | 6 | 5 | 50.4 | 2.6 |
| **37_EPCm** | - | 2015 | 15.8 | -17.34 | 54 | 6 | 6 | 51.9 | 1.5 |
| **37_EPCm** | Y | 2016 | 13.8 | -18.42 | 54 | 6 | 6.5 | 54 | 2.1 |
| **38_EPCm** | - | 2009 | 14.2 | -15.9 | 54 | 6 | 2 | 37.9 | 5 |
| **38_EPCm** | - | 2010 | 15.3 | -16.26 | 54 | 6 | 3 | 44 | 6.1 |
| **38_EPCm** | - | 2010 | 15.4 | -16.5 | 54 | 6 | 3 | 44 | 0 |
| **38_EPCm** | - | 2011 | 16.7 | -17.34 | 54 | 6 | 4 | 49.9 | 5.9 |
| **38_EPCm** | - | 2013 | 15.3 | -18.42 | 54 | 6 | 6 | 53.5 | 1.9 |
| **40_EPCm** | - | 1986 | 12.9 | -14.46 | 62 | 14 | 6 | 47.7 | NA |
| **40_EPCm** | - | 1987 | 14.5 | -14.22 | 62 | 14 | 7 | 53.5 | 5.8 |
| **40_EPCm** | - | NA | 15.1 | -14.82 | 62 | 14 | 7 | 53.5 | 5.8 |
| **40_EPCm** | - | 1989 | 15.6 | -14.94 | 62 | 14 | 9 | 55 | 1.3 |
| **40_EPCm** | - | 1990 | 15.7 | -16.38 | 62 | 14 | 10 | 56.5 | 1.5 |
| **40_EPCm** | - | 1994 | 15.4 | -14.94 | 62 | 14 | 14 | 60.5 | 3.3 |
| **40_EPCm** | Y | 1995 | 15.6 | -14.1 | 62 | 14 | 14 | 62 | 1.5 |
| **41_EPCm** | - | NA | 15.1 | -15.66 | 53 | 5 | 2 | NA | NA |
| **41_EPCm** | - | 2012 | 10.1 | -15.9 | 53 | 5 | 3 | 39.7 | NA |
| **41_EPCm** | - | 2013 | 15.6 | -16.02 | 53 | 5 | 4 | 47.9 | 8.2 |
| **41_EPCm** | - | 2014 | 16.8 | -16.74 | 53 | 5 | 5 | 49.8 | 1.9 |
| **41_EPCm** | Y | 2015 | 16.4 | -17.46 | 53 | 5 | 5 | 53 | 3.2 |
| **42_EPCm** | - | 2009 | 14.3 | -17.1 | 63 | 6 | 1 | 32.8 | NA |
| **42_EPCm** | - | 2010 | 15.5 | -17.58 | 63 | 6 | 2 | NA | 6.3 |
| **42_EPCm** | - | 2011 | 14.8 | -16.5 | 63 | 6 | 3 | 45.4 | 6.3 |
| **42_EPCm** | - | 2012 | 14.5 | -17.7 | 63 | 6 | 4 | 52.9 | 7.5 |
| **42_EPCm** | - | 2013 | 16.3 | -19.74 | 63 | 6 | 5 | 55.6 | 2.7 |
| **42_EPCm** | - | 2014 | 18.5 | -18.66 | 63 | 6 | 6 | 60.9 | 5.3 |
| **42_EPCm** | Y | 2015 | 17.8 | -20.82 | 63 | 6 | 6 | 63 | 2.1 |
| **46_EPCm** | - | 2004 | 14 | -14.7 | 56 | 8 | 1 | 30 | NA |
| **46_EPCm** | - | 2005 | 16.1 | -14.7 | 56 | 8 | 2 | 41.2 | 11.2 |
| **46_EPCm** | - | 2007 | 16.3 | -14.58 | 56 | 8 | 4 | 46.2 | 2.1 |
| **46_EPCm** | - | 2008 | 18.1 | -16.14 | 56 | 8 | 5 | 47.3 | 1.1 |
| **46_EPCm** | - | 2010 | 19.1 | -17.46 | 56 | 8 | 7 | 52.1 | 4.5 |
| **46_EPCm** | - | 2010 | 19 | -16.14 | 56 | 8 | 7 | 52.1 | 4.5 |
| **46_EPCm** | - | 2011 | 18.2 | -17.1 | 56 | 8 | 8 | 55.4 | 3.3 |
| **51_EPCm** | - | 2008 | 16.3 | -16.14 | 57 | 5 | 2 | 33.7 | NA |
| **51_EPCm** | - | 2009 | 17.9 | -16.26 | 57 | 5 | 3 | 46.9 | 13.2 |
| **51_EPCm** | - | 2010 | 18.5 | -16.62 | 57 | 5 | 4 | 54.1 | 7.2 |
| **51_EPCm** | - | 2011 | 19 | -17.22 | 57 | 5 | 5 | 55.7 | 1.6 |
| **51_EPCm** | Y | 2012 | 18.2 | -15.78 | 57 | 5 | 5.5 | 57 | 1.3 |
| **56_EPCm** | - | 2010 | 16.6 | -15.66 | 58 | 7 | 3 | 35.6 | NA |
| **56_EPCm** | - | 2011 | 16.7 | -15.54 | 58 | 7 | 4 | 43.3 | 7.7 |
| **56_EPCm** | - | 2012 | 18.8 | -15.9 | 58 | 7 | 5 | 49.3 | 6 |
| **56_EPCm** | - | 2013 | 16.7 | -13.38 | 58 | 7 | 6 | 52.9 | 3.6 |
| **56_EPCm** | - | 2014 | 15.9 | -16.74 | 58 | 7 | 7 | 57.2 | 4.3 |
